# The Cross-talk Between Intestinal Microbiota and MDSCs Fuels Colitis-associated Cancer Development

**DOI:** 10.1158/2767-9764.CRC-23-0421

**Published:** 2024-04-15

**Authors:** Hadas Ashkenazi-Preiser, Or Reuven, Atara Uzan-Yulzari, Sharon Komisarov, Roy Cirkin, Sondra Turjeman, Carmel Even, Nira Twaik, Kerem Ben-Meir, Ivan Mikula, Leonor Cohen-Daniel, Yaron Meirow, Eli Pikarsky, Yoram Louzoun, Omry Koren, Michal Baniyash

**Affiliations:** 1 The Concern Foundation Laboratories at The Lautenberg Center for Immunology and Cancer Research, Israel-Canada Medical Research Institute, Faculty of Medicine, The Hebrew University, POB 12272, Jerusalem 91120, Israel.; 2 Azrieli Faculty of Medicine, Bar-Ilan University, Ramat Gan, Israel.; 3 Department of mathematics, Bar-Ilan University, Ramat Gan, Israel.

## Abstract

Intestinal chronic inflammation is associated with microbial dysbiosis and accumulation of various immune cells including myeloid-derived suppressor cells (MDSC), which profoundly impact the immune microenvironment, perturb homeostasis and increase the risk to develop colitis-associated colorectal cancer (CAC). However, the specific MDSCs–dysbiotic microbiota interactions and their collective impact on CAC development remain poorly understood. In this study, using a murine model of CAC, we demonstrate that CAC-bearing mice exhibit significantly elevated levels of highly immunosuppressive MDSCs, accompanied by microbiota alterations. Both MDSCs and bacteria that infiltrate the colon tissue and developing tumors can be found in close proximity, suggesting intricate MDSC-microbiota cross-talk within the tumor microenvironment. To investigate this phenomenon, we employed antibiotic treatment to disrupt MDSC–microbiota interactions. This intervention yielded a remarkable reduction in intestinal inflammation, decreased MDSC levels, and alleviated immunosuppression, all of which were associated with a significant reduction in tumor burden. Furthermore, we underscore the causative role of dysbiotic microbiota in the predisposition toward tumor development, highlighting their potential as biomarkers for predicting tumor load. We shed light on the intimate MDSCs-microbiota cross-talk, revealing how bacteria enhance MDSC suppressive features and activities, inhibit their differentiation into mature beneficial myeloid cells, and redirect some toward M2 macrophage phenotype. Collectively, this study uncovers the role of MDSC-bacteria cross-talk in impairing immune responses and promoting tumor growth, providing new insights into potential therapeutic strategies for CAC.

**Significance:**

MDSCs–dysbiotic bacteria interactions in the intestine play a crucial role in intensifying immunosuppression within the CAC microenvironment, ultimately facilitating tumor growth, highlighting potential therapeutic targets for improving the treatment outcomes of CAC.

## Introduction

Chronic inflammation is a common hallmark of various diseases, including infectious diseases, autoimmune disorders, and cancer (1). Over time, sustained inflammatory responses can lose their beneficial effects and create a hostile environment within the host (1). Inflammatory bowel disease (IBD) serves as a prime example of this phenomenon, closely associated with colitis-associated colorectal cancer (CAC; refs. 2, 3). IBD encompasses a group of disorders characterized by chronic intestinal inflammation and disrupted homeostasis, driven by complex interactions between the host's immune system and an evolving microbiota (dysbiosis), ultimately leading to conditions such as cancer (4). Under normal circumstances, the gut microbiota plays a pivotal role in maintaining intestinal homeostasis by regulating immune responses, preventing pathogen colonization, and supporting intestinal epithelial cell function (5, 6). However, environmental factors such as diet, infections, chemicals, and stress can initiate gut inflammation, resulting in dysbiosis and abnormal immune responses. These factors can cause chronic inflammation that damages the mucosal layer, induces DNA aberrations, and promotes epithelial dysplasia, ultimately contributing to CAC development (7).

Numerous studies, including our own, have demonstrated in mice and humans a strong association between prolonged chronic inflammation that features various pathologies including CAC, and the accumulation and activation of myeloid-derived suppressor cells (MDSC; refs. 8–11). MDSCs represent a heterogeneous population of immature myeloid progenitor cells with potent immunosuppressive capabilities. MDSCs can be classified into monocytic (M-MDSC) and polymorphonuclear (PMN-MDSC) subtypes based on surface markers (12, 13). Under chronic inflammatory conditions, MDSC precursors in the bone marrow (BM) with basal suppressive activity, expand and differentiate into immunosuppressive cells that migrate to inflamed sites and periphery, promoting immunosuppression (14). MDSCs exert their immune-suppressive activity through various mediators, including nitric oxide (NO), reactive oxygen species (ROS), and secreted cytokines, leading to dysfunction in immune cells such as T and natural killer (NK) cells (15, 16). Importantly, this immunosuppression can be reversed with appropriate anti-inflammatory treatments affecting MDSCs and/or by eliminating the inflammation causing stimuli, thus restoring the normal function of the immune system that ensues in disease regression (17).

Given the accumulating evidence that uncontrolled intestinal chronic inflammation leads to colitis characterized by dysbiosis and an altered immune response, we hypothesized that a cross-talk between dysbiotic microbiota and recruited MDSCs exacerbates inflammation and immunosuppression, thus supporting CAC development. Our study demonstrates the intricate interactions between invading bacteria and recruited MDSCs in the damaged intestine during CAC. Antibiotic treatment effectively disrupts this MDSC-bacteria cross-talk, leading to reduced MDSC levels and suppressive activity, thereby alleviating intestinal and peripheral inflammation and resulting in tumor regression. We also elucidate the causal role of bacteria in predisposing the host to a higher tumor burden and their potential as predictive biomarkers for tumor development. In addition, we uncover the mechanisms by which dysbiotic bacteria affect MDSCs, intensifying the inflammatory and immunosuppressive environment conducive to CAC development and progression.

Our findings shed light on the complex interplay between intestinal microbiota and MDSCs during chronic inflammation, providing fundamental insights into the pathogenesis of colitis and its progression to CAC. This research highlights potential predictive tools and therapeutic strategies for disease prevention and treatment.

## Materials and Methods

### Mice

C57BL/6J female mice were purchased from Envigo (RRID:MGI:7260344) at 8–10 weeks of age were used in all experiments to minimize variability related to gender and age differences, ensuring reproducibility. The mice were purchased from Harlan and were grown at the Hebrew University specific pathogen-free facility and maintained on a 12 hours light/dark cycle, food, and water *ad libitum*. For the fecal microbiota transplantation (FMT) experiments alone, Swiss Webster germ free (GF) mice were grown at Bar-Ilan University (Ramat Gan, Israel) and then transferred to the Hebrew University facility and maintained under the same conditions. All mice were fed with Teklad Rodent Diet (irradiated global 18% protein fixed formula), and acclimated for 14 days before the induction of CAC. In addition, each experiment contained at least two cages for each group to avoid cage effect. Control mice were untreated and kept in the animal facility under the same conditions and for the same duration as the CAC-induced mice.

### Induction of CAC

Initially, mice were injected intraperitoneal with 10 µg/mouse Azoxymethane (AOM; Sigma, A5486). Seven days later, mice received 2% dextran sodium sulfate (DSS; MP-Bio, 160110) dissolved in autoclaved tap water for 5 days followed by 16 days of autoclaved tap water. The DSS cycles were repeated for total of three cycles. Mice were monitored daily for the appearance of clinical symptoms such as weight loss, diarrhea, and rectal bleeding. Two to 3 weeks after the last cycle of DSS, mice were sacrificed and the blood, spleen, BM, and colon tissues were collected and analyzed. Fecal samples were collected at different timepoints.

### Hematoxylin and Eosin Tissue Histology

Colon and spleen tissues were fixed with 4% formalin, embedded in paraffin and sectioned with a microtome to 7 µm sections. Tissues were stained with hematoxylin and eosin (H&E) solution and observed using an Olympus BX51 Fluorescence Microscope (RRID:SCR_018949); image acquisition was with Olympus cellSens Software (RRID:SCR_014551). The H&E slides underwent blinded examination by a pathologist who quantified the adenomas, assessed their grade, and determined the numbers of intramucosal and invasive carcinomas.

### Immunofluorescence and FISH

Colon and spleen tissues were embedded in optimal cutting temperature compound (Scigen O.C.T. Compound Cryostat Embedding Medium, 23-730-625) and sectioned in a cryostat to 7 µm sections. Sections were then fixed with 4% paraformaldehyde (PFA), blocked using 3% BSA, 3% goat serum, and 0.03% Tween-20 and stained with antibodies against CD3ε (FITC) CD11b (FITC), Ly6C (PE), and Ly6G (Alexa Fluor 647). For FISH staining, slides were incubated in FISH staining buffer (0.9 mol/L NaCl, 20 mmol/L Tris-HCl, 0.01% SDS, 20% Formamide) containing 2 mmol/L Alexa Fluor 647–conjugated bacterial 16S probe EUB338 (5-GCTGCCTCCCGTAGGAGT-3) for 2 hours at 46°C and washed with washing buffer (215 mmol/L NaCl, 20 mmol/L Tris-HCl, and 5 mmol/L Ethylenediaminetetraacetic acid (EDTA)) for 15 minutes at 48°C as described previously (18). Subsequently, sections were stained with (Thermo Fisher Scientific, D1306) and mounted using ProLong Gold mounting medium (Thermo Fisher Scientific, P10144). Images were taken using an Olympus microscope and an Olympus IX 81 Inverted Fluorescence Automated Live Cell Microscope (RRID:SCR_020341).

### Gut Permeability Assay

At the endpoint of the experiments, mice were deprived of food and water for 1 hour. Then, mice were given 10 mg/mouse FITC-dextran (Sigma, FD4) diluted in sterile PBS by oral gavage. Four hours later, mice were sacrificed and blood was collected. Blood was kept at 4°C at least for 1 hour and then centrifuged for 10 minutes at 1,000 × *g* to separate the serum. A total of 100 µL of serum sample was loaded in 96-well flat black plates and fluorescence intensity was measured at a wavelength of 480 nm excitation and 535 nm emission using a Spark 20M (Tecan).

### Tissue Harvest and Generation of Single-cell Suspensions

Peripheral blood, spleen, BM, and colon were excised from control and CAC mice. Then, the spleens were mashed in PBS, resuspended in erythrocyte lysis buffer (ELB; 150 mmol/L NH_4_Cl, 10 mmol/L KHCO_3_, 0.1 mmol/L EDTA), washed in PBS and filtered through a 70-µm strainer. BM cells were flashed out using a syringe, resuspended in ELB, washed in PBS and filtered through a 70-µm strainer. Blood was collected and resuspended in ELB, washed in PBS and filtered through a 70 µm strainer. Preparation of single-cell suspension from colon tissues was performed as previously described with mild modification (19). Fecal content was washed from colons, which were then opened longitudinally and cut into 0.5 cm pieces. The pieces were shaken in 20 mL Hank's Balance Salt Solution (HBSS) buffer containing 10 mmol/L HEPES, 2 mmol/L EDTA, and 1 mmol/L Dithiothreitol (DTT) for 20 minutes at 37°C and vortexed, and then the supernatant was collected, filtered through a 70 µm strainer and kept on ice. This step was repeated one more time. Then, the remaining pieces were digested in 5 mL Ca2+ Mg2+ HBSS buffer containing 10 mmol/L HEPES, 5% FCS, 1% Penicillin/streptomycin (pen-strep), 1 mg/mL collagenase D (Sigma, C5138), and 0.1 mg/mL DNase I (Sigma, 11284932001) for 30 minutes in a shaker at 37°C. The supernatant was collected, filtered through a 70 µm strainer and kept on ice. The remaining tissue was mashed on top of a 70 µm cell strainer and washed to collect any further detached cells. All fractions were combined and centrifuged at 500 × *g* for 10 minutes at 4°C to collect the isolated cells. Then, the cells were washed twice in cold PBS, filtered through a 40 µm strainer and resuspended in PBS containing 1.5% FCS for further use.

### Flow Cytometry and Antibodies

Cells were stained with antibodies against CD11b (FITC, Pacific Blue or APC; M1/70. RRID: AB_312788, AB_755985, AB_312794), Ly6C (FITC, PE, or Alexa Fluor 700; HK1.4. RRID: AB_1186134, AB_1186132, AB_10640119), Ly6G (Alexa Fluor 647 or biotin; 1A8. RRID: AB_1134159, AB_1186105), CD3ε (APC; 145–2C11. RRID: AB_312676), Thy1.2 (APC; 30-H12. RRID: AB_313182), MHCII (FITC, M5/114.15.2. RRID: AB_2621715), CD86 (PE; PO3. RRID: AB_313158), CD11c (PE/Cy7; N418. RRID: AB_493569), F4/80 (APC; BM8. RRID: AB_893493), CD206 (Brilliant Violet 421; C068C2. RRID: AB_2562232), and CD45.2 (APC or Alexa Fluor700; 104. RRID: AB_389210, AB_493730), in combination with unlabeled anti-CD16/32 (clone 93. RRID: AB_312800). All antibodies were acquired from BioLegend, except those against MHC II (Tonbo). All cell surface staining was performed in PBS containing 1.5% FCS and 0.05% sodium azide (FACS buffer), on ice for 30 minutes and then washed with FACS buffer. For intracellular staining of CD247, cells were fixed in 1% PFA in PBS for 20 minutes on ice, permeabilized with 0.1% saponin (Sigma, S4521) in PBS for 10 minutes in room temperature and stained with APC or PE-conjugated anti-CD3ε and FITC-conjugated anti-CD247 (H146) in the presence of anti-CD16/32 in FACS buffer containing 0.1% saponin for 30 minutes on ice. Data acquisition was performed on a Beckman Coulter CytoFLEX Flow Cytometer (RRID:SCR_019627) and data were analyzed using FCS express v8 (DeNovo software).

### Isolation and Purification of PMN-MDSCs and M-MDSCs

PMN-MDSCs were enriched from splenocytes and BM using a magnetic column separation system (Miltenyi Biotec). Cells were pooled from 6 to 8 mice per group. Briefly, cells were first labeled with anti-Ly6G conjugated to biotin (30 minutes at 4°C), then washed and labeled (45 minutes at 4°C) with streptavidin magnetic microbeads (Miltenyi Biotec 130-048-101), washed and loaded onto a column placed in the magnetic field. The positive (PMN-MDSC) and negative cell populations were isolated according to the manufacturer's directions (Miltenyi Biotec). M-MDSCs were enriched from the negative cell population using the EasySep Monocyte Isolation kit (StemCell Technologies, 19861) according to the manufacturer's instructions. Then, the cells were stained for Ly6G, Ly6C, CD11b, and CD45.2 in PBS containing 1.5% FCS and 2 mmol/L EDTA. Ly6G−Ly6ChiCD11b+ (M-MDSCs) and Ly6G+Ly6ClowCD11b+ (PMN-MDSCs) were sorted using FACS ARIA III (BD). Single-cell suspensions from colons were prepared as described above and directly stained for sorting. Samples were counted using a TC20 automated cell counter (Bio-Rad). All cell populations were above 90% purity.

### *Ex Vivo* T-cell Proliferation Inhibition Assay

A total of 96-well (U-shape) plates were precoated with 100 µL of 0.1 mol/L Borate buffer (pH = 8.5) containing 3 µg/mL anti-CD3ε (BioLegend, 145-2C11. RRID: AB_2616674), 3 µg/mL anti-CD28 (BioLegend, 37.51. RRID: AB_2810333) for 24 hours at 4°C. Wells were then washed with full medium [RPMI1640 (Gibco, 21875-034) containing 8% FCS (Sigma, F7524), 50 µmol/L β-2-mercaptoethanol (Gibco, 31350-010), 2 mmol/L l-glutamine (Biological Industries, 03-020-1C), and 1% pen-strep solution, Biological Industries, 03-031-1C]. T cells were isolated from normal spleens using EasySep Mouse T Cell Isolation Kit (StemCell Technologies,19851) according to the manufacturer's instructions and stained with carboxyfluorescein diacetate succinimidyl ester (CFSE; Thermo Fisher Scientific, C1157) as described previously (17). A total of 1 × 10^5^ CFSE-labeled T cells were resuspended in full medium, seeded in each well and M-MDSCs or PMN-MDSCs were added on top of the T cells at different T:MDSC ratios. Cocultures were kept in the incubator, at 37°C for 72 hours. In some experiments, M-MDSCs were preincubated with bacteria for 1 hour before adding them to the wells. Samples were harvested in cold FACS buffer and stained with anti-Thy1.2-APC (BioLegend). The proliferation index was determined with FCS-Express V6 proliferation analyzer (DeNovo software).

### *Ex Vivo* CD247 Downregulation Assay

A total of 1 × 10^5^ T cells per well were seeded in 96-well (U-shape) plates and M-MDSCs or PMN-MDSCs were added on top of the T cells at different T:MDSC ratios. Cocultures were kept in the incubator, at 37°C for 18 hours. Cells were harvested in cold PBS and stained for CD247 as described above. Expression levels of CD247 were measured using mean fluorescence intensity (MFI) by flow cytometry.

### Punch Biopsy

Pieces of mouse colon tissue were excised using 3 mm biopsy puncher and incubated at 37°C overnight in 100 µL RPMI containing 8% FCS, 10 mmol/L HEPES 2 mmol/L l-glutamine, and 1.5% pen-strep. The supernatant was then collected, centrifuged for 10 minutes at 1,000 × *g* to discard debris and kept at −20°C until further analysis.

### Bacterial DNA Extraction, Amplification, and Sequencing

Fecal/colon tissue samples for microbiota analysis were collected from mice at different timepoints during the experiments and kept at −20°C until use. DNA was extracted using the Power Soil DNA Isolation Kit (MoBio) according to the manufacturer's instructions, following a bead beating step (BioSpec) for 2 minutes. Next, extracted DNA was used for PCR amplification of the V4 variable region of the bacterial 16S rRNA gene by primers, 515F- (barcoded) 5′-AATGATACGGCGACCACCGAGATCTACACGCTAGCCTTCGTCGCTATGGTAATTGTGTGYCAGCMGCCGCGGTAA-3′ and 806R 5′-CAAGCAGAAGACGGCATACGAGATAGTCAGTCAGCCGGACTACHVGGGTWTCTAAT-3′ (20) PCR reactions were carried out with the Primestar Taq polymerase (Takara) for 30 cycles of denaturation (95°C), annealing (55°C) and extension (72°C), and final elongation at 72°C. Products were purified using AMPure magnetic beads (Beckman Coulter), and quantified using the Picogreen double-stranded DNA (dsDNA) quantitation kit (Invitrogen). Samples were then pooled at equal amounts, loaded on 2% agarose E-Gel (Thermo Fisher Scientific) and purified using NucleoSpin Gel and PCR Clean-up (Macherey-Nagel). Purified products were sequenced using the Illumina MiSeq platform (Genomic Center, Azrieli Faculty of Medicine, BIU, Israel).

### 16S rRNA Gene Sequence Analysis

FASTQ data were processed and analyzed using the Quantitative Insights into Microbial Ecology 2 (QIIME2) pipeline version 2019.4 (21). Single-end sequences were first demultiplexed using the q2‐demux plugin. To improve taxonomic resolution, reads were denoised and clustered using DADA2 via q2‐dada2 (22). MAFFT (23) and FastTree2 (24) were used for alignment and phylogeny construction for all amplicon sequence variants using q2‐alignment and q2‐phylogeny plugins, respectively. Taxonomy classification was done using q2‐feature‐classifier (25), while final feature sequences were aligned against Greengenes database with 99% confidence (26). To avoid any possible contamination, the feature table was filtered via q2-feature-table. First, features that were annotated as mitochondria and chloroplast were filtered out; next, features that were found in less than three samples were filtered out. For the GF experiment, low abundance features (<0.0005%) were also filtered out from the feature table.

The analysis was performed on a rarefied table > 10,000 reads per sample. α diversity was calculated using the Faith's Phylogenetic Diversity (PD; ref. 27) measure, referring to bacterial richness within the sample, and significant differences in bacterial richness between the groups were determined using the Kruskal–Wallis test. β diversity was analyzed according to weighted (quantitative) and unweighted (qualitative) UniFrac distances (28) to compare differences in gut bacterial communities between the sample groups. To evaluate the level of significant permutational multivariate analysis of variance (PERMANOVA) test was performed, as implanted in QIIME2 with the default of 999 permutations for both weighted and unweighted UniFrac. Significant differences in bacterial abundance between control and CAC groups were examined at the genus level using the discrete FDR test for microbiota samples from the last day of the experiment. dsFDR was calculated using the mean-rank test statistics with an FDR threshold of qm > 0.1 (29). The analysis and Heatmap visualization were created using Calour (30).

### FMT to GF Mice and Sample Collection

Fecal samples for FMT were collected from CAC and control mice at the last timepoint of the experiment and kept at −20°C until use. To obtain three pools of FMT donors for each group, CAC and control fecal samples, nine fecal pellets from different mice were collected and divided into three pools randomly. Each pool was suspended in 3 mL sterile PBS and dissolved by vortex for 1 minute. A total of 200 µL of the fecal suspension was administered by oral gavage to Swiss Webster GF female mice 8–10 weeks old (control ≥7 and CAC ≥9). Transplanted GF mice were housed in isolated cages and maintained on 12 hours light/dark cycle at the Hebrew University animal facility.

### Microbiome Preprocessing, Machine Learning, and Correlations

#### Preprocess

We preprocessed the 16S rRNA gene sequences of each dataset using the MIP-MLP pipeline (31). The preprocessing of MIP-MLP contains three stages: merging similar features based on the taxonomy, scaling the distribution and standardization to z scores. We merged the features at the genus taxonomy by mean before using all the models. We performed log normalization as well as z-scoring on the mice. No dimension reduction was used at this stage.

#### Machine Learning

In the first experiment, we used only the CAC-bearing mice group. Mice with final tumor load above the median were given a tag of 1, and mice with less than median were given a tag of 0. To predict whether the final tumor load of a mouse is above or below the median based on its initial microbiota, we used a random forest classifier with the default parameters and 14 estimators. The results of learning are an average of 20 different runs of a leave one out learning (one sample is in the test each time). Our metric was AUC. In the FMT experiment we took the CAC-induced groups and predicted which mice will develop tumors at the final timepoint. We used a random forest classifier with the defaulting parameters and seven estimators. The results of learning are an average of 20 different runs of a leave one out learning (one sample is in the test each time). Our metric was AUC.

#### Correlations

After the preprocess was done, we considered only the CAC bearing mice group and tried to find correlated bacteria at T0 to the final tumor load. Our metrics were R2 as well as Spearman correlation coefficient. We calculated the correlation between the amount of each bacterium at T0 and the final tumor load of each mouse within the CAC group. The taxonomy tree displays the taxonomy levels which are correlated with the final tumor load. The value of each of the leaves is set to be its correlation with the final tumor load and the value of an internal node is the mean of its direct descendants in the tree. We set the Spearman correlation threshold as 0.5. Every node with higher value was painted in blue and nodes with a value lower than −0.5 were painted in red in the taxonomy tree. In the FMT experiment, we performed a similar analysis. We considered all the CAC-induced mice and calculated the correlation between the amount of each bacterium at T1 and the final tumor at the end of the experiment. Again, we introduce our taxonomy tree based on the FMT experiment correlations.

For the detection of bacteria associated with a condition, we used a nonparametric permutation test. Reported correlations have higher absolute values of correlation coefficient than 99% of all correlation coefficients obtained in scrambled samples, where the bacterial load was scrambled over all samples.

### Isolation of Bacteria from Fecal Samples

Fecal pellets from 3 to 5 mice per group (control and CAC mice) were collected, homogenized in 10 mL sterile PBS and filtered through 100 µm to exclude large debris following centrifugation for 3 minutes at 100 × *g* to exclude small debris. Then, bacteria were pelleted by centrifugation for 10 minutes at 3,000 × *g*. The bacterial cells were washed twice in PBS, aliquoted and frozen at −20°C until use. On the day of the experiment, the bacterial cells were resuspended in RPMI containing 8% FCS and 2 mmol/L l-glutamine. Quantification of bacterial cells was done by optical density readings (600 nm), where an optical density (O.D.) of 1 was considered equivalent to 1 × 10^9^ bacterial cells. To confirm that the numbers of bacteria between samples were similar, DNA from the bacterial pellets was extracted and RT-PCR for the 16S rRNA region was performed.

### ImageStream Analysis

Single-cell suspension of total BM was generated using the protocol described above. Cells were then stained with antibodies against Ly6C (PE) and Ly6G (Alexa Fluor 647). Bacteria pellets produced from fecal samples of control or CAC mice were stained with CFSE at a concentration of 0.625 µmol/L. Antibody-labeled cells and CFSE-labeled bacteria were subsequently cocultured for 45 minutes at 37°C and fixed with 1% PFA. Cells were then washed with PBS, permeabilized using 0.1% saponin and stained for DNA content using DAPI. ImageStream (Amnis) was used for data acquisition and image collection. Data analysis was done using IDEAS software. Statistics represents means of samples.

### Differentiation Assay

Immature monocytes were isolated from BM of normal mice as described above. A total of 1 × 10^5^ monocytes were resuspended in RPMI (Gibco) containing 8% FCS, 10 mmol/L HEPES, 2 mmol/L l-glutamine and seeded in 96-well flat-bottom plates and isolated bacteria from fecal samples were added on top of the cells at a ratio of 1:100. Two hours later, 1% pen-strep and 20 ng/mL of GMCSF (PeproTech, 315-03) or 20 ng/mL MCSF (PeproTech, 315-02) were added and the cocultures were kept in the incubator at 37°C for 72 hours. Cells were harvested using Accutase solution (Sigma, A6964), washed and stained with anti MHC II (FITC), anti CD86 (PE), anti F4/80 (APC) and anti CD206 (BV421) in FACS buffer as described above.

### Coculture of MDSCs and Bacteria

A total of 2.5 × 10^5^ isolated immature monocytes were resuspended in RPMI containing 8% FCS, 2 mmol/L l-glutamine, and 10 mmol/L HEPES and seeded in 96-well U-shape plates, and isolated bacteria from fecal samples were added on top of the cells at 1:100 M-MDSCs:bacteria ratio, unless indicated otherwise. Two hours later, pen-strep Solution (Bio industries, 03-031-1C) was added to final concentration of 1% and the cocultures were kept in the incubator for 18 hours. Then, the plates were centrifuged, the supernatants were collected and frozen at −20°C for further use and the cells were washed in PBS, pelleted and kept frozen at −20°C until further use.

### NO Detection

Cell-free supernatants from M-MDSCs:bacteria cocultures were mixed with 1 mg/mL Griess reagent (Merck, K39713137) dissolved in 5% phosphoric acid at 1:1 ratio. A total of 100 µL of this mixture was loaded in ELISA plates and absorbance was measured at wavelength of 540 nm using Spark 20M (Tecan).

### Arginase Activity

M-MDSCs pellets from M-MDSCs:bacteria cocultures were lysed in 50 µL 0.1% Triton-X-100 (Sigma, X100) containing 1 ng/mL protease inhibitor (Millipore, 539134) for 30 minutes at room temperature. After lysis, 50 µL of 25 mmol/L Tris-HCL (pH 7.5) and 5 µL of 10 mmol/L MnCl_2_ were added and the lysate was incubated for 10 minutes at 56°C for enzyme activation. A total of 100 µL of 0.5 mol/L, pH9.7, l-arginine (Sigma, A5131) was added to the activated lysate, and this mix was incubated for 2 hours at 37°C. The reaction was stopped by adding 900 µL of acidic mixture [9% sulphuric acid (V/V) and 27% phosphoric acid (V/V)]. Then, 40 µL of 9% ISPF solution (Sigma, I3502) dissolved in ethanol was added. This mixture was heated for 30 minutes at 95°C. A total of 100 µL of each sample was loaded in an ELISA plate and absorbance was measured at wavelength of 540 nm using Spark 20M (Tecan).

### Detection of ROS Production in PMN-MDSC

A total of 2.5 × 10^5^ isolated immature PMN-MDSCs were resuspended in RPMI containing 8% FCS, 10 mmol/L HEPES, 2 mmol/L l-glutamine and the cells were seeded in 96-well U-shape plates. Isolated bacteria from fecal samples were added at 1:50 PMN-MDSCs:bacteria ratio. Then, DCFDA 2′,7′-Dichlorofluorescin diacetate (H2DCFDA, Merck, D6883) dissolved in RPMI was added to final a concentration of 10 µmol/L. The cocultures were incubated for 4 hours at 37°C. Without washing, cells were analyzed by flow cytometry using CytoFLEX. ROS production was measured by flow cytometry, using the MFI of the 488 laser.

### Measurements of Cytokine and Chemokine Levels

Protein levels of IL1α, IL1β, IL6, IL10, IL12p70, IL17A, IL23, IL27, MCP-1, IFNβ, IFNγ, TNFα, and GMCSF were determined in cell-free supernatants using the LEGENDplex Mouse Inflammation Panel (13-plex, LEGENDplex, BioLegend, 740150) according to the manufacturer's instruction. Data were collected using CytoFLEX and analyzed using The LEGENDplex Data Analysis Software.

### Antibiotic Treatment

AOM/DSS-treated mice were given a cocktail of three antibiotics dissolved in autoclaved tap water after the third cycle of DSS until the end of the experiment (2–3 weeks). The antibiotics used were 0.5 g/L imipenem (Gerda), 1 g/L metronidazole (B. Braun), and 0.5 g/L vancomycin (Mylan). The antibiotic cocktail was replaced every 3 days.

### Statistical Analysis

Unpaired, nonparametric Student *t* tests (Mann–Whitney) with one-tailed distributions were performed to examine statistical differences between two experimental groups. When body weight and tumor volume were investigated, two-way ANOVA was performed. The analysis was performed on GraphPad Prism 8.0 software (Graph Pad Software, Inc.). Data are presented as mean ± SEM. *P* values lower than 0.05 were considered significant. In the figures, *, *P* < 0.05; **, *P* < 0.01; ***, *P* < 0.001; ***, *P* < 0.0001. Statistics regarding microbiota analysis are described in “16S rRNA gene sequence analysis.”

### Study Approval

Animal use followed protocols approved by the Hebrew University- Hadassah Medical School Institutional Animal Care and Use Committee.

### Data Availability Statement

The data generated in this study are available upon request from the corresponding author.

## Results

### Intestinal and Peripheral Inflammation in CAC Mice is Associated with Increased MDSC Levels and Immunosuppression

**FIGURE 1:**
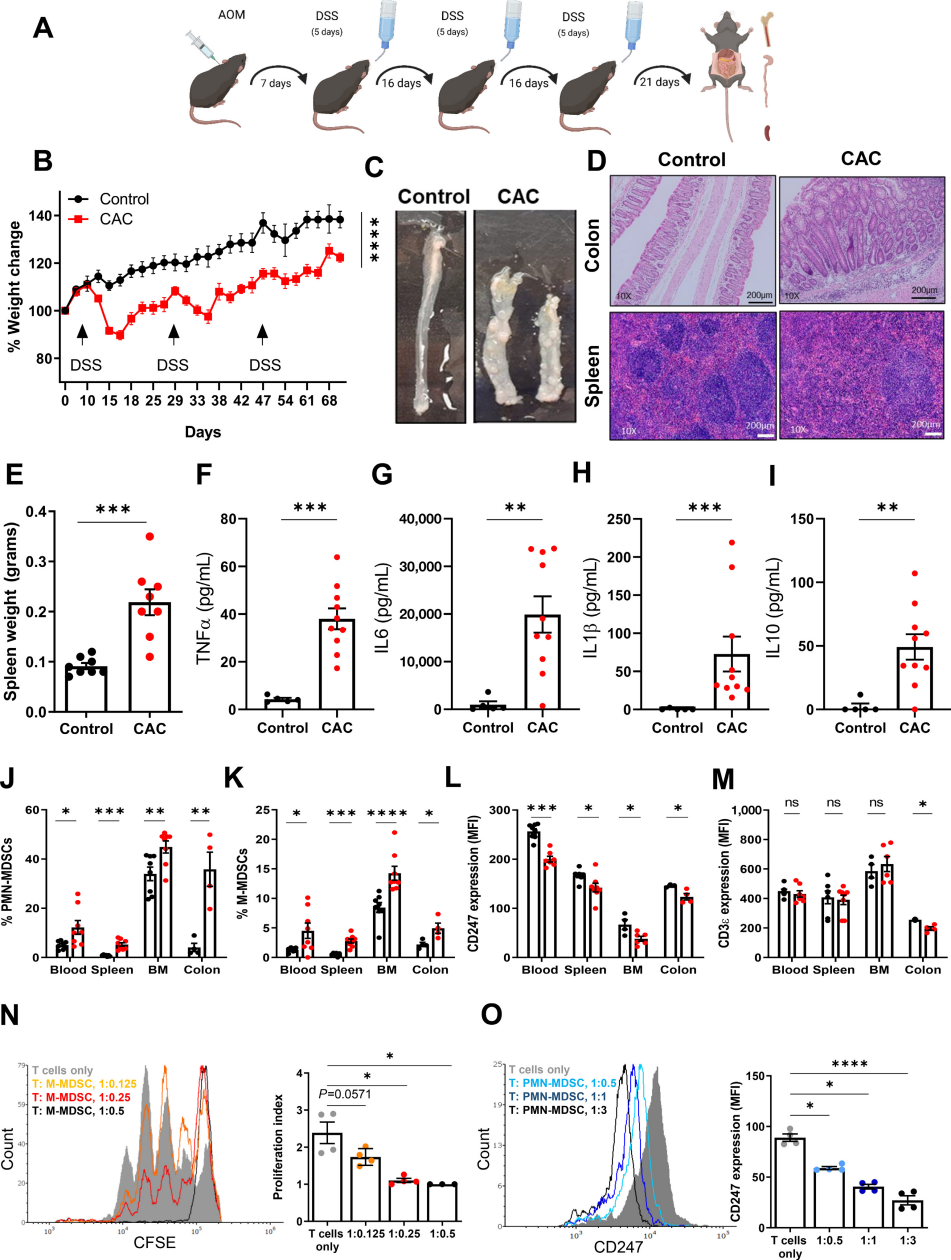
CAC-bearing mice display site specific and systemic inflammation during disease progression. **A,** Schematic presentation of the AOM/DSS-based CAC model used. Created with BioRender.com. **B,** Weight change during disease progression, normalized to the start point (*n* = 8 per group). **C,** Representative images of colons. **D,** H&E staining of the colon (top) and spleen (bottom) tissue sections of control and CAC mice; Magnification: 10x, Scale bar: 200 µm. **E,** Spleen weights (*n* = 8). Levels of TNFα (**F**), IL6 (**G**), IL1β (**H**), and IL10 (**I**) in supernatants of cultured colon punch biopsies (*n* = 5 or 10). In **J**–**M**, black dots represent the control group and red dots represent the CAC group. Flow cytometry data showing percentages of PMN-MDSCs (**J**) and M-MDSCs (**K**) in the blood, spleen, BM, and colon. Cell percentages were calculated from total cell populations. **L,** Flow cytometry data showing expression levels of CD247 (presented by MFI) in CD3ε+ T cells in blood, spleen, BM, and colon (*n* = 3–8). **M,** Flow cytometry data showing expression levels of CD3ε (presented by MFI) in blood, spleen, BM, and colon (*n* = 3–8). Expression levels of CD247 were calculated from CD3^+^ cells. Representative data from one out of three independent experiments. **N,** Representative histograms illustrate the proliferation of activated T cells cocultured with different ratios of M-MDSCs isolated from the spleens of CAC mice (left). The corresponding proliferation index is summarized on the right (*n* = 4). **O,** Representative histograms of CD247 expression levels in naïve T cells cocultured with different ratios of PMN-MDSCs isolated from spleen of CAC mice (left). The corresponding CD247 levels are summarized as MFI on the right (*n* = 4). Results are represented as mean ± SEM. *, *P* < 0.05; **, *P* < 0.01; ***, *P* < 0.001; ****, *P* < 0.0001.

In this study, we investigated the relationship between MDSC-mediated immunosuppression, microbial dysbiosis, and tumor development during chronic intestinal inflammation. We employed the AOM/DSS mouse model to induce CAC that recapitulates the human disease histopathology (Fig. 1A; ref. 32). As our previous studies shown similarity in MDSC levels and functions between female and male mice across different models of chronic inflammation, we chose to use female mice of the same strain and age in this study. This facilitated attainment of reproducible results with reduced variability. The CAC mice in the presented model exhibited symptoms of intestinal inflammation, including diarrhea and weight loss (Fig. 1B). At the experiment's endpoint, 3 weeks after the last DSS cycle, we then sacrificed and analyzed the mice. The colons of CAC mice were shorter than those of age-matched control mice, indicating developing intestinal inflammation. Macroscopic examination (Fig. 1C) and histologic analysis (Fig. 1D, top) revealed growing tumors in the colon, associated with a pronounced inflammatory and reactive state, characterized by the presence of infiltrating leukocytes and the formation of enlarged epithelial lymphoid structures (Supplementary Fig. S1A). Tumor-bearing mice had enlarged spleens (Fig. 1E) with distorted morphology (Fig. 1D, bottom), as reflected by the domination of the red pulp, leading to distortion in the germinal center architecture when compared with control spleen sections. Such a splenic distortion is associated with elevated levels of recruited MDSCs as indicated by flow cytometry analyses (Fig. 1J and K). These findings indicated severe site-specific (colon) and peripheral (spleen) inflammation in CAC mice, accompanied by increased levels of inflammation-related cytokines detected in colon punch biopsies, including TNFα (Fig. 1F), IL6 (Fig. 1G), IL1β (Fig. 1H), and IL10 (Fig. 1I). Additional cytokines such as IL1α, IL12p70, IL17A, IL23, IL27, MCP-1, IFNβ, IFNγ, and GMCSF were also upregulated in the culture media of colon punch biopsies from CAC mice compared with control mice (Supplementary Fig. S2A).

We next compared MDSC levels between CAC and control mice in the colon and periphery. Flow cytometry analyses revealed elevated levels of PMN- and M-MDSCs in the blood, spleen, BM, and colon of CAC mice compared with control mice (Fig. 1J and K). To assess the immune status of CAC mice, we examined the expression levels of the CD247 molecule (TCR ζ chain) in T cells from different tissues, as it serves as a biomarker for T-cell receptor (TCR) integrity. We observed downregulated CD247 levels in T cells from the blood, spleen, and BM (Fig. 1L) of CAC mice compared with control mice, with unchanged CD3ε levels (Fig. 1M), indicating an immune-suppressed state of T cells. In the colon, CD247 levels were downregulated to a greater extent compared with CD3ε (Fig. 1L and M). Immunofluorescence analyses of colon sections confirmed elevated levels of MDSCs within the inflamed tumor-bearing colons (Supplementary Fig. S2B).

To assess the functional characteristics of the two MDSC subpopulations, PMN- and M-MDSCs were isolated from the spleens of CAC mice and cocultured with naïve T cells activated in vitro through the TCR and CD28. M-MDSCs from the spleen of CAC mice were able to inhibit T-cell proliferation in a T:M-MDSC ratio-dependent manner (Fig. 1N). In contrast, PMN-MDSCs did not significantly affect T-cell proliferation even when cocultured with T cells in a higher ratio (Supplementary Fig. S3A). However, coculturing naïve T cells with PMN-MDSCs from spleens of CAC mice led to downregulated CD247 levels in T cells, with the effect increasing with the T:PMN-MDSC ratio (Fig. 1O), while M-MDSCs induced only a mild CD247 downregulation when cocultured with T cells in a higher ratio (Supplementary Fig. S3B). CD3ε expression levels remained unchanged (Supplementary Fig. S3E), indicating an immunosuppressed state of T cells rather than activation. These results demonstrate distinct roles of MDSC subpopulations in modulating T-cell responses *in vivo* during tumor development and highlight the association between site-specific (colon) and peripheral (blood, spleen) chronic inflammation and an elevated immunosuppressive environment.

### CAC Mice Exhibit Alterations in Microbiota Diversity and Composition

**FIGURE 2:**
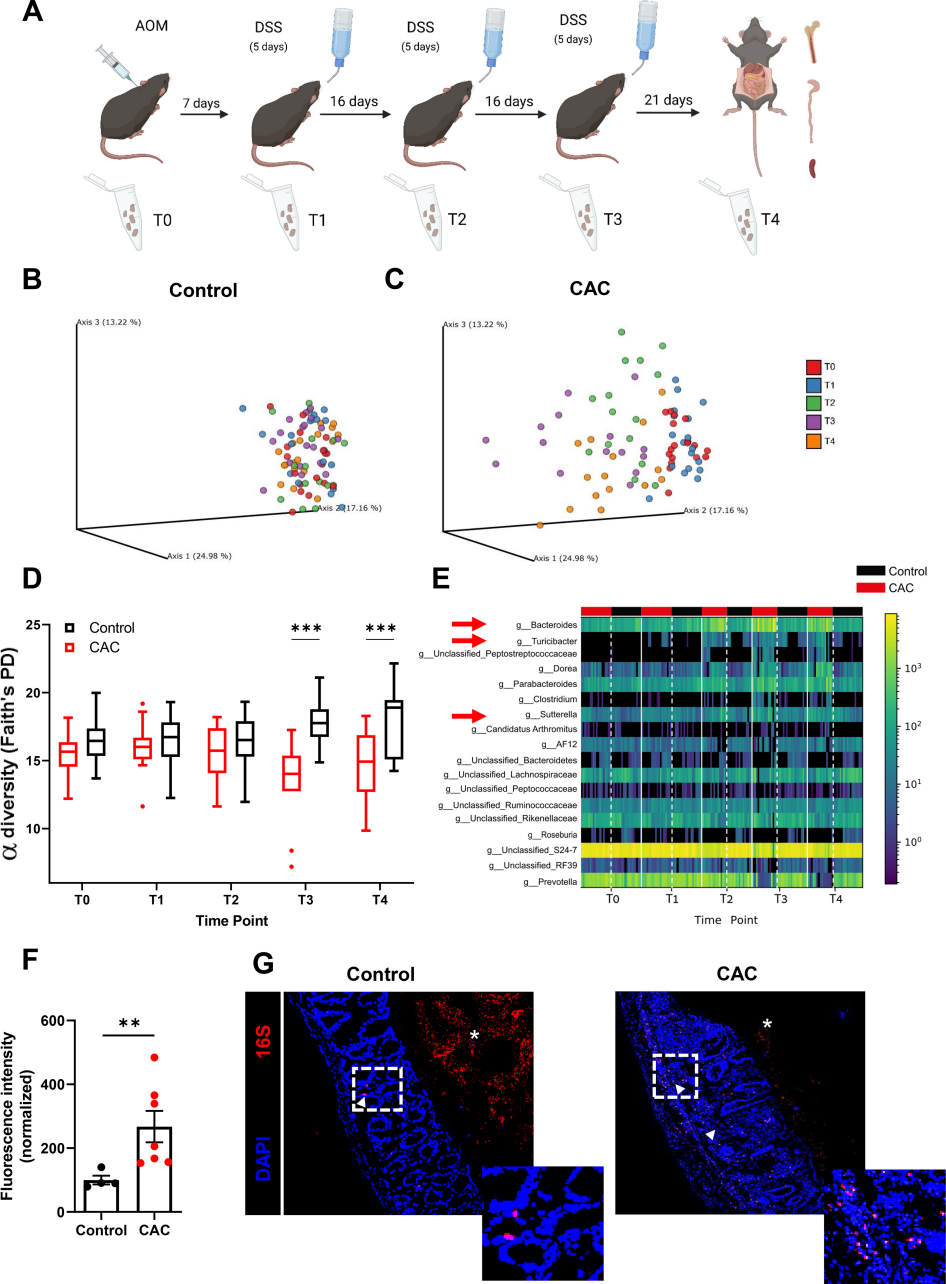
CAC-bearing mice display altered microbiota composition and diversity. **A,** Schematic presentation of different timepoints for fecal samples collection. Created with BioRender.com. PCoA plots showing microbiota composition analysis of control (**B**) and CAC (**C**) mice at the different timepoints during the experiment. **D,** α diversity measured using Faith's PD. **E,** Heat map based on Calour, depicting changes in bacteria between the groups throughout the experiment (overrepresented bacteria are marked with red arrows). White dashed lines separate the CAC and control groups at each timepoint. **F,** FITC-dextran fluorescence intensity in serum normalized to control mice (*n* = 4–7 per group). **G,** FISH staining of bacteria in colonic tissue sections of CAC-bearing mice compared with control mice. White arrows indicate bacterial penetration into the tissue. Asterisks indicate the lumen; Magnification: 10x, (*n* = 3). Magnification: 20x, Scale bar, 50 µm. Inserts, represent a magnification of the white dashed squares. Results are represented as mean ± SEM; **, *P* < 0.01; ***, *P* < 0.001.

Previous studies have demonstrated that intestinal chronic inflammation is associated with changes in gut microbiota composition (33). To investigate whether the CAC mice in this study exhibited altered microbiota compared with control mice, we collected fecal samples at different timepoints during disease progression (Fig. 2A) and assessed microbiota composition using 16S rRNA gene sequencing. Principal coordinates analysis (PCoA) revealed that the microbiota of control mice remained relatively stable throughout the experiment (Fig. 2B). In contrast, CAC mice showed a noticeable shift in bacterial community composition during the course of colitis-associated tumor development (Fig. 2C).

When we compared bacterial richness between the groups (α diversity, Faith's PD), we found significantly lower diversity among CAC mice in the later stages of disease progression compared with control mice (Fig. 2D, timepoints T3 and T4). Analysis of differential abundance at the endpoint identified 18 significantly different genera between the groups. Notably, Bacteroides, Sutterella, and Turicibacter were relatively elevated in the CAC group (Fig. 2E).

Next, we evaluated the integrity of colon tissue in CAC mice by measuring the fluorescence intensity of FITC-dextran in the serum following its administration via gavage. As shown (Fig. 2F), CAC mice exhibited increased gut permeability compared with control mice. These results correlated with FISH staining of colon sections, which indicated the presence of dislocated bacteria invading the colon tissue of CAC mice compared with control mice (Fig. 2G). These findings suggest that dysbiosis and the infiltration of bacteria into damaged colon tissue may contribute to the creation of an environment that supports CAC development.

### FMT from CAC Mice Supports Tumor Development

**FIGURE 3:**
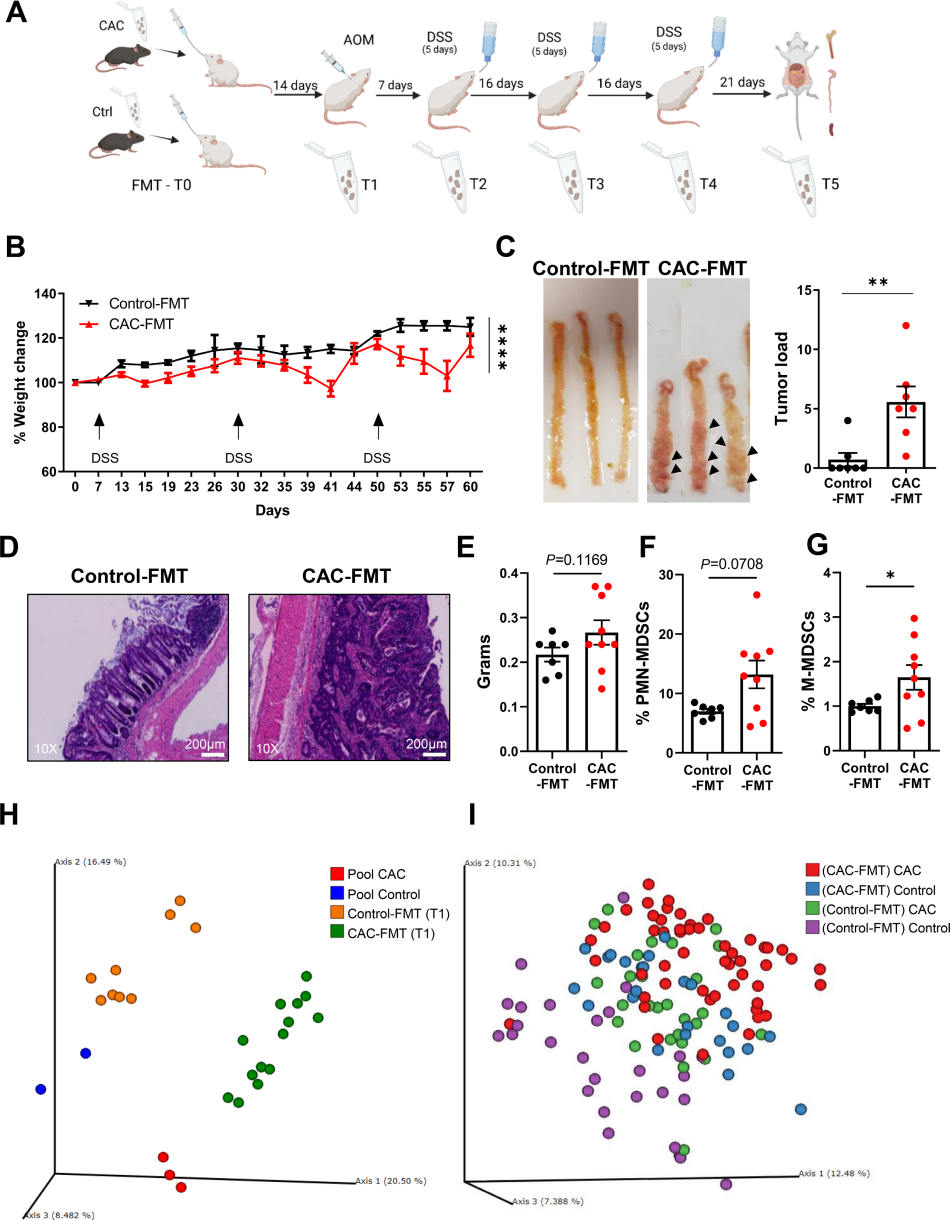
CAC-associated microbiota promotes tumor development. **A,** Schematic presentation of CAC/FMT experiment and fecal sample collection. Created with BioRender.com. **B,** Weight change in CAC-induced mice during disease progression (*n* = 7–9). Weights are normalized to the starting point. **C,** Representative images of colons (left) and summarized tumor load (right). Black arrowheads represent macroscopic tumors. **D,** Representative H&E staining of colon sections. **E,** Spleen weights. Flow cytometry data showing the percentages of PMN-MDSCs (**F**) and M-MDSCs (**G**) in the spleens (*n* = 7–9). **H,** PCoA plots showing microbiota composition of control (*n* = 2) and CAC (*n* = 3) donor samples (pools) and fecal samples of recipient mice 14 days after microbiota transfer (T1). **I,** PCoA plots showing microbiota composition analysis of control and CAC-induced control-FMT and CAC-FMT mice at all timepoints (T1–T5) during the course of the experiment. Results are presented as mean ± SEM; *, *P* < 0.05; **, *P* < 0.01.

We proceeded to investigate whether the altered microbiota composition associated with CAC could support tumor development and growth in CAC-induced mice by influencing MDSC levels and immunosuppressive environment. To address this, GF mice were first subjected to FMT from CAC (CAC-FMT) or control (control-FMT) mice. Both FMT donor pools exhibited differences in their microbiota composition (Supplementary Fig. S4A), consistent with the microbiota presented in Fig. 2E. After microbiota colonization (day 15), the FMT recipients, including CAC-FMT and control-FMT mice, underwent the CAC model induction or were left untreated (Fig. 3A). CAC-induced CAC-FMT mice developed a severe pathology compared with the CAC-induced control-FMT mice, as evidenced by significant weight loss (Fig. 3B), higher tumor incidence (Fig. 3C), and H&E staining of the colon tissues (Fig. 3D). While CAC-FMT CAC mice did not have significantly larger spleens as compared with control-FMT CAC mice (Fig. 3E), they contained increased levels of PMN-MDSCs (Fig. 3F) and M-MDSCs (Fig. 3G). Microbiota establishment was confirmed in mice that underwent FMT using 16S rRNA gene sequencing and β diversity analysis through PCoA. As expected, we found convergence of both the CAC-FMT mice and control-FMT mice with the respective inoculation pools after 15 days (T1; Fig. 3H). When examining the microbiota community and comparing between the different groups during the course of the experiment, we observed that CAC mice of the CAC-FMT and control-FMT groups clustered closely (Fig. 3I), whereas they were in separate clusters initially (Fig. 3H). In line with our previous results, Sutterella and Turicibacter were more abundant in CAC (control-FMT and CAC-FMT) mice at T5 as compared with T1 (Supplementary Fig. S4B). However, the outcome regarding tumor development differed between the groups, with CAC mice from the CAC-FMT group developing significantly more tumors than those in the control-FMT group (Fig. 3C). Taken together, these results show that tumor development is influenced not only by the evolving chronic inflammatory environment but also by the initial microbiota composition, highlighting the interplay between these factors in shaping CAC pathogenesis.

### Predisposition and Predictive Association Between the Microbiota Composition Prior to CAC Induction and Tumor Load

**FIGURE 4:**
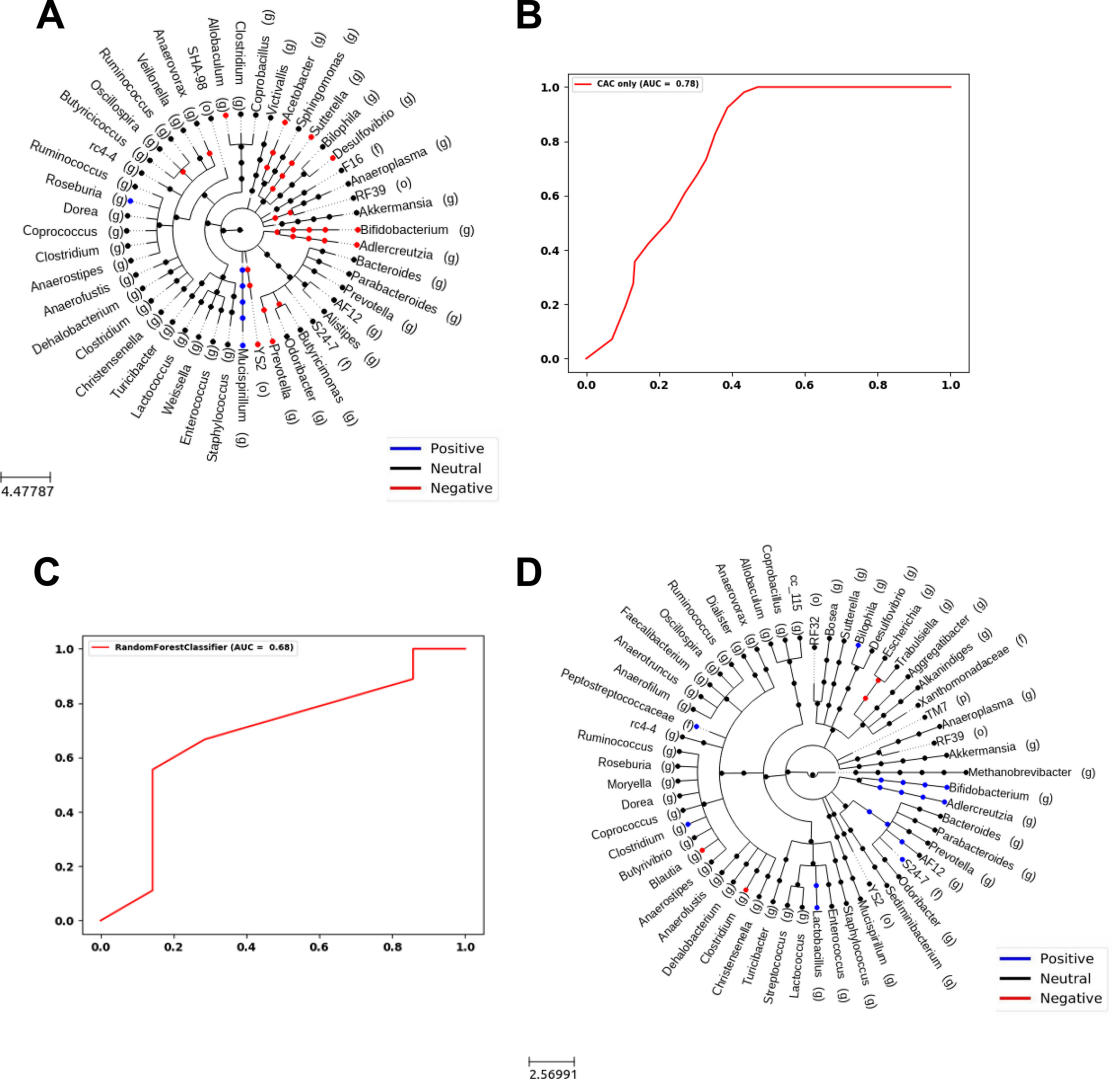
Initial microbiota composition correlates with tumor load and predicts tumor development. **A,** Taxonomy tree of bacteria correlated with high tumor load. Blue points are significant positive correlations, red points are significant negative correlations and black points are bacteria in the sample uncorrelated with the tumor load. **B,** ROC curve of tumor load prediction. The input was the microbiome at T0 and the prediction was whether the final tumor load was below or above the median. **C,** ROC curve of tumor load prediction in CAC-induced samples of control-FMT and CAC-FMT mice together. The input was the microbiome at T15, the prediction was whether the final tumor load was below or above the median. **D,** Taxonomy tree of bacteria correlated with high tumor load in control-FMT and CAC-FMT mice. Blue points are significant positive correlations, red points are significant negative correlations, and black points are bacteria in the sample uncorrelated with the tumor load.

We further tested whether the microbiota composition present prior to CAC induction predisposes the host to enhanced tumor development. To explore this, we capitalized on the observed wide variation in tumor load among the CAC mice (Fig. 1), and categorized these mice into groups of high and low/no tumor load, based on dissections performed at the end of the experiment. High and low tumor loads were defined as above and below median tumor load in the CAC group, respectively. We computed the correlation between the bacterial composition at the initial timepoint and the tumor load. Several genera were detected as associated with the endpoint tumor load, including a negative correlation of Prevotella, YS2, Allobaculum, Acetobacter, Sutterella, Desulfovibrio, Bifidobacterium, and Adlercreutzia and a positive correlation of Roseburia and Mucispirillum (Fig. 4A). To assess whether the initial microbiota composition (prior to CAC induction) could predict high versus low tumor load, we applied an XGBoost classifier to the initial microbiome data. We found that the final tumor load could be predicted with an AUC of 0.78 (Fig. 4B). Thus, the initial microbiota is not only associated with the final tumor load, but can also serve as a predictive biomarker for tumor development. This prediction may result from a causal relationship between the initial microbiota and tumors or could be a bystander effect.

We then applied the same XGBoost on the CAC-induced CAC-FMT and control-FMT mice presented in Fig. 4, and found that the groups could be predicted with an AUC of 0.68 (Fig. 4C). Blautia and a certain Clostridium species exhibited negative correlations with CAC, while genera such as Bifidobacterium, Adlercreutzia, Bilophila, S24-7, a different Clostridium species than mentioned above and Lactobacillus, showed positive correlations with the outcome (Fig. 4D). These findings show that the bacteria composition at the initial stage, before CAC induction, not only dictates the tumor load after CAC induction but can also serve as predictive markers for tumor development.

### Microbiota Depletion Reduces Colon and Peripheral Inflammation, Resulting in Low Tumor Incidence

**FIGURE 5:**
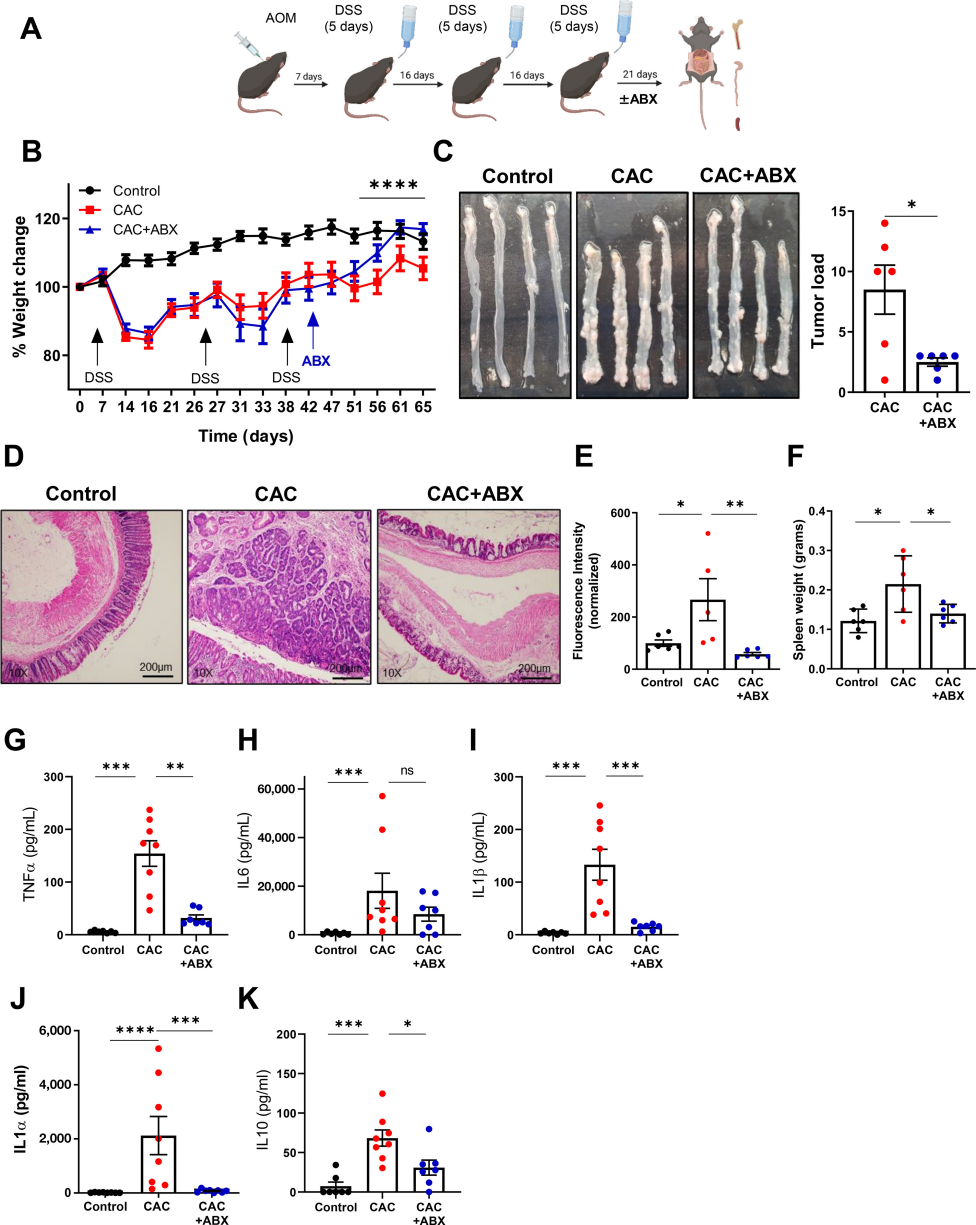
Microbiota depletion in CAC-bearing mice reduces colon inflammation and tumor load. **A,** Schematic presentation of CAC/ABX experiment and fecal sample collection. Created with BioRender.com. **B,** Weight change of control, CAC, and ABX-treated CAC mice during disease progression normalized to mice weight at the start point (*n* = 6). Statistical analysis was performed comparing between CAC and ABX-treated CAC mice from day 51 to day 65. **C,** Representative images of colons. Tumor load is summarized (right; *n* = 6). **D,** Representative H&E-stained colon section. Magnification: 10x, Scale bars: 200 µm. **E,** FITC-dextran fluorescence intensity in serum normalized to control mice (*n* = 5–6). **F,** Spleen weights (*n* = 6). Levels of TNFα (**G**), IL6 (**H**), IL1β (**I**), IL1α (**J**), and IL10 (**K**) in supernatants of cultured colon punch biopsies (*n* = 6–8). Data representing two independent experiments. Results are represented as mean ± SEM; *, *P* < 0.05; **, *P* < 0.01; ***, *P* < 0.001.

Our findings, which demonstrate MDSC accumulation in the colon and periphery in association with dysbiosis and bacteria invasion into the colon tissue, prompted us to investigate whether depletion of the microbiota will affect MDSC features and functions, ultimately leading to the restoration of the immune environment and an improved disease outcome. To explore this, CAC mice were treated with a cocktail of antibiotics (ABX) after the third cycle of DSS (Fig. 5A), a disease stage that is featured by enhanced inflammation and established tumors as indicated by the H&E analysis (Supplementary Fig. S5A–S5C). The combination of antibiotics was employed to achieve a comprehensive depletion of the microbiota. Analysis of the microbiota profile following ABX treatment revealed a shift in the bacterial composition in ABX-treated CAC mice, resulting in a significant decrease in bacterial diversity and complete domination of the family ABX-resistant Enterobacteriaceae (Supplementary Fig. S6A–S6C), along with a reduction in the total bacterial load among them of the genera Bacteroides, Sutterella, and Turicibacter (Supplementary Fig. S6D). The ABX treatment had several beneficial effects on CAC mice compared with the ABX-untreated CAC mice, as evidenced by: (i) Regained body weight (Fig. 5B), (ii) Recovery of colon macroscopically (Fig. 5C) and histologically (Fig. 5D) as indicated by H&E stain (Supplementary Fig. S5A), (iii) A significant reduction in tumor load (flat and polypoid) as indicated macroscopically (Fig. 5C) and by histologic quantification (Supplementary Fig. S5A–S5C), (iv) Restoration of colon epithelial integrity to a normal condition, as indicated by reduction in colon permeability (Fig. 5E). This was further confirmed by FISH staining, which showed reduced levels of bacteria invading the colon tissue in ABX-treated CAC mice as compared with ABX-untreated CAC mice (Supplementary Fig. S6E), (v) Reduction in spleen size (Fig. 5F), suggesting inflammation relief not only in the colon but also in the periphery, and (vi) Decreased secretion of proinflammatory cytokines such as TNFα, IL1β, IL1α, and IL6 and the anti-inflammatory cytokine IL10 (Fig. 5G and K) from colon tissue punch biopsies of ABX-treated CAC mice compared with ABX-untreated CAC mice. Other inflammation-related cytokines such as, IL12p70, IL17A, IL23, IL27, MCP-1, IFNβ, IFNγ, and GMCSF showed similar levels in the culture media of colon punch biopsy of ABX-treated and ABX-untreated CAC mice (Supplementary Fig. S7). These results collectively demonstrate that microbiota depletion results in a reduced colon and peripheral inflammation, leading to tumor regression, in association with restoration of the colonic structure and improved overall disease outcomes in CAC mice.

### Antibiotic Treatment of CAC Mice Results in Reduced MDSC Levels and Suppressive Functions in the Colon and Periphery

**FIGURE 6:**
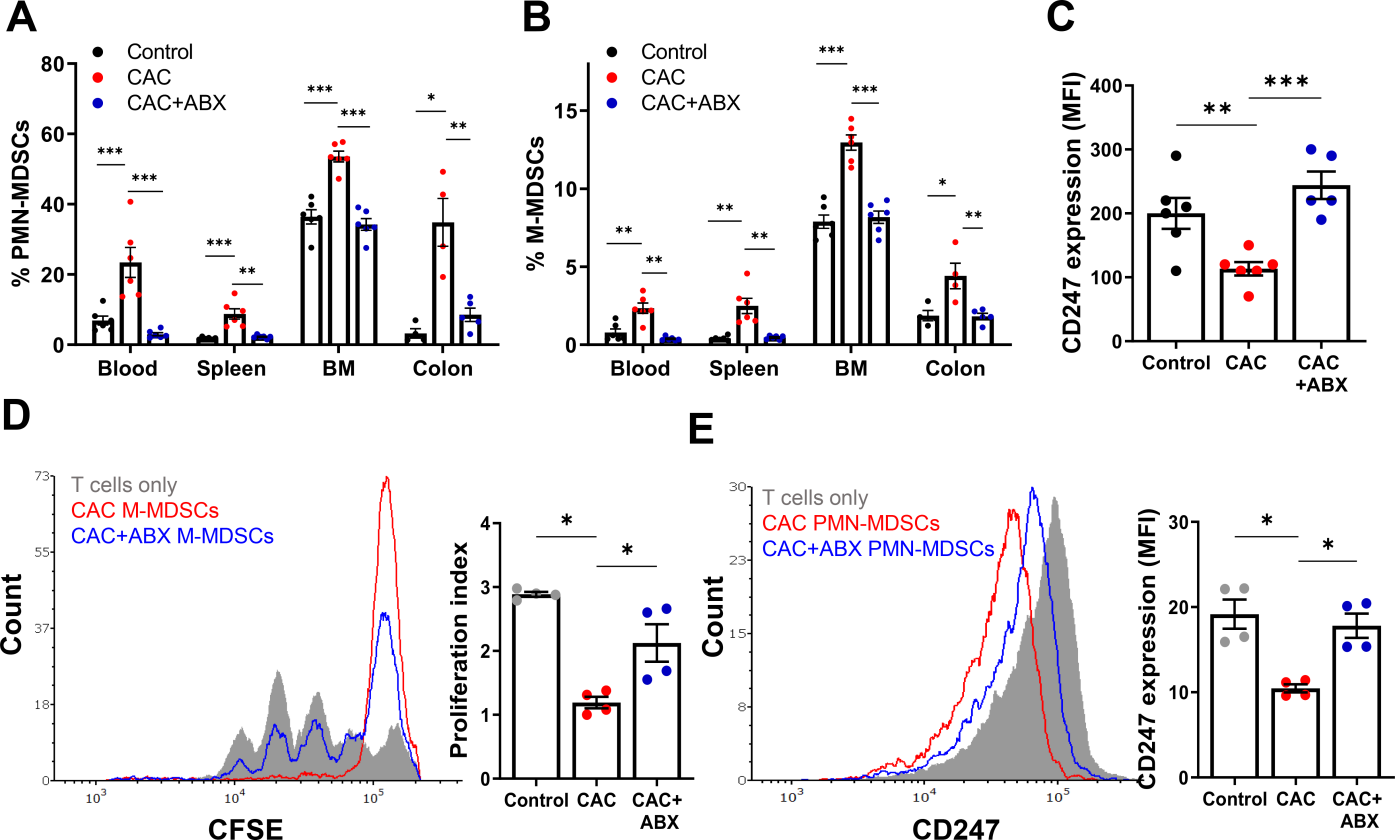
Microbiota depletion in CAC-bearing mice reduces MDSCs levels and their suppressive function. Flow cytometry data showing the percentages of PMN-MDSCs (**A**) and M-MDSCs (**B**) in the blood, spleen, BM, and colon (*n* = 4–6). Cell percentages were calculated from the total cell populations. **C,** Flow cytometry analysis showing CD247 expression levels in splenic T cells (*n* = 6), calculated from CD3^+^ cells. Representative data from one of three independent experiments. **D,** Proliferation of activated T cells that were cocultured with M-MDSCs isolated from spleens of CAC or ABX-treated CAC mice at a ratio of 1:0.25. Representative histograms (left) and the summarized proliferation index (right) are presented (*n* = 4). **E,** Expression levels of CD247 in naïve T cells cocultured with PMN-MDSCs isolated from the spleen of CAC and ABX-treated CAC mice at a ratio of 1:1 is depicted. Representative histograms (left) and summarized MFI (right) are shown (*n* = 4). Data represent two independent experiments. Results are represented as mean ± SEM; *, *P* < 0.05; **, *P* < 0.01; ***, *P* < 0.001.

We proceeded to investigate the effects of the ABX treatment on MDSC levels and their suppressive features and functions in both the intestine and periphery. Our findings revealed a significant decrease in the levels of both PMN- and M-MDSCs in the blood, spleen, BM, and colon (Fig. 6A and B, respectively). The observed decrease in MDSC levels in ABX-treated CAC mice correlated with the recovery of CD247 expression levels in T cells (Fig. 6C). We then assessed the suppressive function of the MDSC subpopulations isolated from the different experimental groups. Interestingly, M-MDSCs isolated from ABX-treated CAC mice exhibited reduced suppressive activity compared with M-MDSCs isolated from the ABX-untreated CAC mice, as evidenced by their impact on T-cell proliferation (Fig. 6D). Similarly, when evaluating the effects of ABX treatment on PMN-MDSC suppressive activity, we observed that PMN-MDSCs isolated from ABX-treated CAC mice induced less CD247 downregulation compared with PMN-MDSCs from ABX-untreated CAC mice (Fig. 6E). These results collectively indicate that ABX treatment not only prevented further bacterial invasion but also led to reduced inflammation, decreased MDSC levels, and suppressed MDSC functions. This reduction in MDSC-mediated immunosuppression, coupled with tumor regression, suggests that dysbiotic bacteria in CAC mice contribute to the establishment of a perpetuated inflammatory environment that supports CAC development.

### The Cross-talk Between MDSCs and CAC-associated Microbiota Enhances Their Suppressive Features and Functions

**FIGURE 7:**
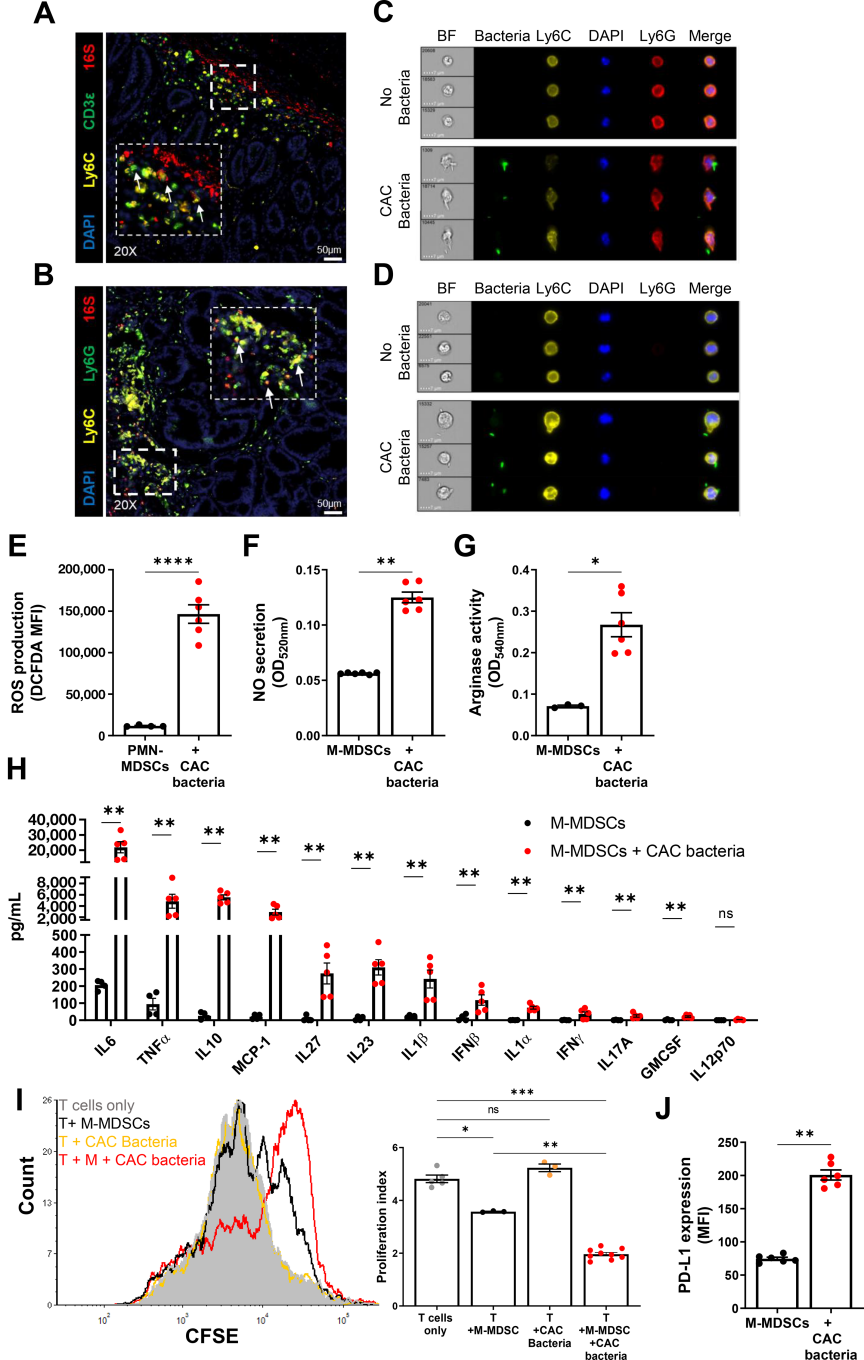
MDSC–bacteria interactions enhance the cells’ suppressive features and functions. **A,** FISH staining of bacterial 16S rRNA (red) combined with immunofluorescence staining of CD3ε+ T cells (green) and ly6C+ M-MDSCs (yellow) were performed on colon sections of CAC-bearing mice. Arrows indicate interactions between M-MDSCs and bacteria (*n* = 2–3 per group). Magnification: 20x, Scale bar, 50 µm. **B,** FISH staining of bacterial 16S rRNA (red) combined with immunofluorescence staining of Ly6G+ PMN-MDSCs (green) and ly6C+ M-MDSCs (yellow) was performed on colon sections of CAC-bearing mice. Arrows indicate interactions between MDSCs and bacteria (*n* = 2–3 per group). Magnification: 20x, Scale bar, 50 µm. Representative images of ImageStream analysis of PMN-MDSCs (**C**) and M-MDSCs (**D**) incubated with or without bacteria isolated from fecal samples of CAC mice at ratio of 1:50 (MDSC:bacteria; *n* = 4–6). **E,** Intracellular ROS production by isolated PMN-MDSCs following incubation with or without bacteria, measured by DCFDA MFI using flow cytometry (*n* = 4–6). **F,** Secretion of NO by isolated M-MDSCs following incubation with or without bacteria, measured by Griess reagent (*n* = 6). **G,** Arginase activity of isolated M-MDSCs following incubation with or without bacteria (*n* = 3–6). **H,** IL6, TNFα, IL10, MCP-1, IL27, IL23, IL1β, IFNβ, IL1α, IFNγ, IL17A, GMCSF, and IL12p70 levels in the supernatants of M-MDSCs following incubation with or without bacteria (*n* = 4–5). **I,** Proliferation of activated T cells cocultured with M-MDSCs that were preincubated with or without bacteria at ratio of 1:100, or T cells cocultured with bacteria at the same ratio. Representative histograms are shown (left), and proliferation index is summarized (right; *n* = 4–6). **J,** Expression levels of PDL-1 in M-MDSCs following incubation with or without bacteria (*n* = 6). Representative data of one out of two independent experiments. Results are represented as mean ± SEM; *, *P* < 0.05; **, *P* < 0.01; ****, *P* < 0.0001.

On the basis of the presented results, which indicate that during CAC development, MDSCs are recruited to the colon in parallel to the invading altered microbiota, we hypothesized that an intimate MDSC–bacteria interaction within the colon tissue is responsible for the heightened MDSC suppressive activities, creating an environment conducive to tumor growth. Indeed, combining immunofluorescence and FISH staining of colon sections from CAC-bearing mice for the detection of MDSCs and bacteria, respectively, revealed that MDSCs and bacteria are found in a close proximity *in vivo* (Fig. 7A and B). To assess the direct effects of the bacteria population enriched in the colon of CAC mice on MDSCs, pools of bacteria were isolated from fecal samples and incubated *in vitro* with precursor MDSCs isolated from normal BM. These precursor cells were chosen for their minimal suppressive features and functions, allowing us to identify which pathways in MDSCs are triggered by the bacteria and whether they can induce MDSCs to become highly suppressive and proinflammatory. Our analysis showed that PMN-MDSCs and M-MDSCs formed a direct contact with the bacteria as visualized by ImageStream analysis, and in some cases, the bacteria were engulfed by the cells (Fig. 7C and D). Furthermore, PMN-MDSCs altered their morphology, as indicated by the reduced circularity score (Supplementary Fig. S8A). M-MDSCs lost their circularity as well but to a lesser extent (Supplementary Fig. S8B). These findings indicate that MDSCs have the ability to make a physical contact with bacteria, which could further impact on MDSC features and functions to become more suppressive. To delve deeper into the pathways activated in MDSCs by bacteria, we examined the response of precursor M-MDSCs and PMN-MDSCs when cocultured with bacteria isolated from fecal samples of CAC mice. Exposure of PMN-MDSCs to bacteria significantly increased ROS production (Fig. 7E), while M-MDSCs increased secretion of NO and levels of arginase activity when cocultured with bacteria (Fig. 7F and G, respectively). These factors produced by MDSCs, in response to bacteria, underlie their suppressive features. Coincubation of M-MDSCs with bacteria resulted in the secretion of high levels of proinflammatory cytokines such as IL6, TNFα, and MCP-1 and the anti-inflammatory cytokine IL10 (Fig. 7H). To assess the ability of bacteria to prime MDSCs toward suppressors of T cells, M-MDSCs were initially incubated with bacteria from fecal samples of CAC mice. Subsequently, they were cocultured with naïve T cells activated through the TCR and CD28. Bacteria-primed M-MDSCs exhibited a more potent inhibitory effect on T-cell proliferation than nonprimed control M-MDSCs. Importantly, the bacteria themselves did not exert a direct effect on the T cells (Fig. 7I). Moreover, bacteria-primed M-MDSCs upregulated the expression of the inhibitory molecule PD-L1 (Fig. 7J), further supporting the suppressive features of bacteria-triggered M-MDSCs.

**FIGURE 8:**
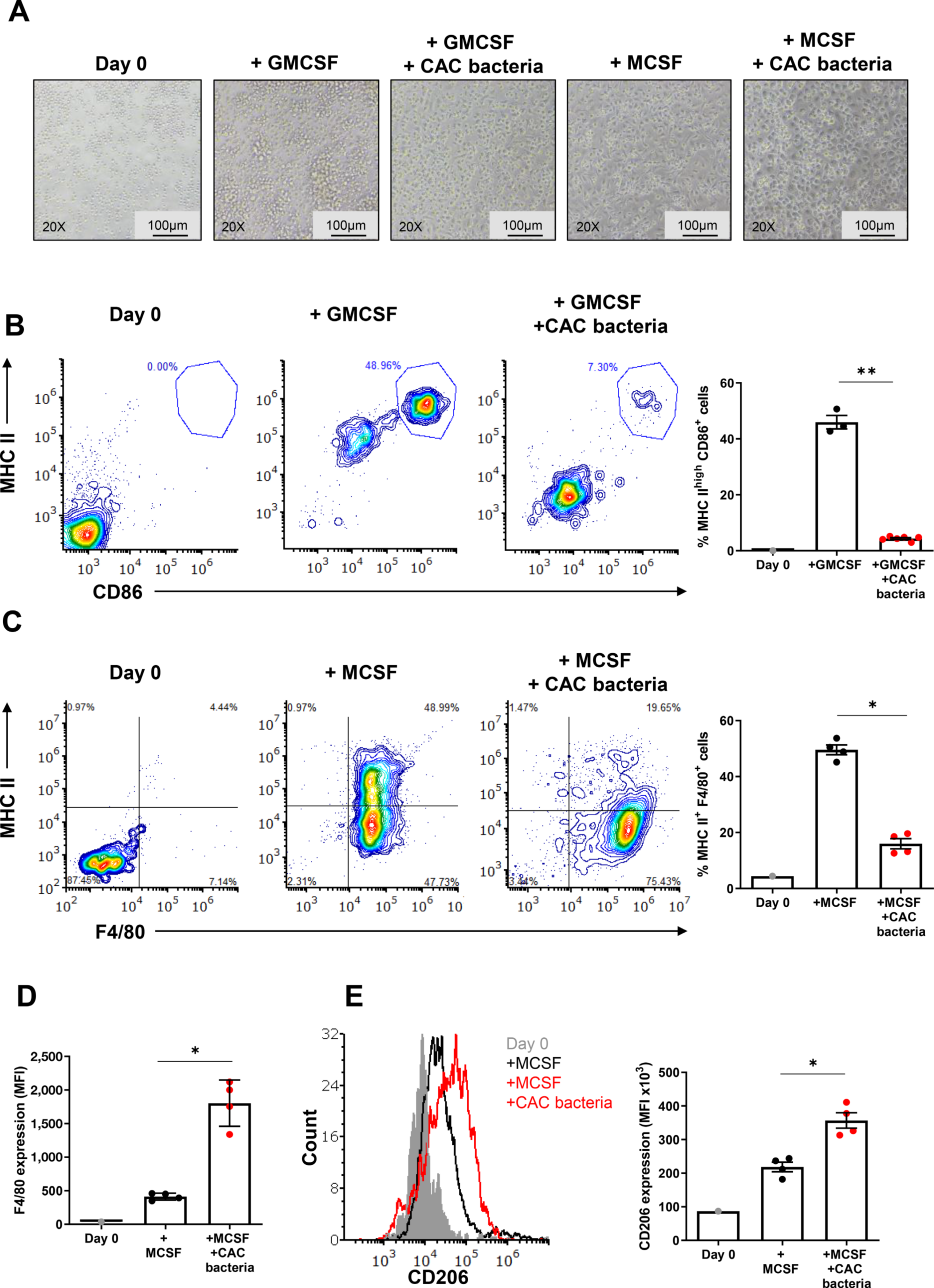
Bacteria polarize M-MDSCs toward immunosuppressive M2 phenotype. **A,** Representative microscopic images of cell cultures of M-MDSCs at day 0 and after 72 hours incubation with or without bacteria at a ratio of 1:100, in the presence or absence of GMCSF or MCSF (*n* = 3–6). Magnification: 20x, Scale bar, 100 µm. **B,** Flow cytometry plots of the surface markers MHC II and CD86 of M-MDSCs (left). MHC II+ CD86^+^ cells are gated and their percentages are summarized (right; *n* = 4–6). **C,** Flow cytometry plots of the surface markers MHC II and F4/80 of M-MDSCs. MHC II+ F4/80+ cells are gated and their percentages are summarized in (right; *n* = 4–6). **D,** Flow cytometry analysis of F4/80 expression in M-MDSCs that were incubated in the presence or absence of bacteria at a ratio of 1:10 with or without MCSF (*n* = 4 per group). Results are represented as MFI. **E,** Flow cytometry histogram showing the expression levels of CD206 in M-MDSCs that were incubated with or without bacteria at a ratio of 1:10, in the presence or absence of MCSF (left; *n* = 4). Results are summarized and the MFI is presented (right). Representative data of one out of two independent experiments. Results are represented as mean ± SEM; *, *P* < 0.05; **, *P* < 0.01.

As mentioned previously, M-MDSCs are immature cells that typically differentiate into effector cells such as macrophages and dendritic cells under normal conditions. We were interested in understanding the effect of CAC bacteria on the differentiation of M-MDSCs. Surprisingly, M-MDSCs stimulated with GMCSF or MCSF, which would normally induce differentiation into mature myeloid cells like granulocytes, neutrophils, dendritic cells, and macrophages, exhibited distinct behavior in the presence of bacteria. M-MDSCs did not respond to GMCSF nor changed their morphology in response to MCSF when bacteria were present (Fig. 8A). Furthermore, M-MDSCs cocultured with bacteria did not upregulate differentiation and maturation markers such as MHC class-II and CD86 in response to GMCSF or MCSF stimulation (Fig. 8B and C, respectively), indicating differentiation arrest induced by the bacteria. However, it was observed that M-MDSCs upregulated F4/80 expression, a macrophage marker, in response to bacteria stimulation (Fig. 8D), which raised the question of whether CAC bacteria may alter M-MDSC maturation toward the immunosuppressive M2 phenotype. Indeed, we found that M-MDSCs upregulated CD206 expression, a marker for M2 macrophages, upon coculture with CAC bacteria in the presence of MCSF (Fig. 8E). These changes align with the enhanced proinflammatory and inhibitory cytokines secreted by MDSCs upon coculture with the bacteria (Fig. 7H). Thus, exposure of MDSCs to CAC bacteria can induce differentiation arrest of M-MDSCs, keeping them in their immature and suppressive state and potentially skewing them to mature, immunosuppressive phenotype resembling M2 macrophages. Indeed, analysis of the bacteria residing within tumor samples isolated from CAC mice revealed that the genera Sutterella and Bacteroides, which are found in the fecal samples of the CAC mice (Fig. 2), also colonize the tumors (Supplementary Fig. S8C). These genera were found alongside other genera including Lactobacilius and Bacteroidales. The presented results strongly suggest that CAC development, within the damaged colon and tumors, recruited MDSC subpopulations and the invading bacteria closely interact, thus amplifying the cells’ suppressive features and functions. These interactions perpetuate the local tumor inflammatory microenvironment, which can strongly support tumor progression and continued damage to the colon tissue.

## Discussion

In the current study, we focused on the interrelation between two key players, MDSCs and intestinal microbiota, which are involved in the process of CAC development during intestinal chronic inflammation, as shown by numerous studies including our own in mice and humans (8–11). By utilizing a mouse model for CAC that mimics the human pathology, we observed peripheral and site-specific chronic inflammation. We detected enlarged spleens, abnormal morphology of the colon, an increase in colon permeability and augmented levels of proinflammatory cytokines such as TNFα, IL6, IL1β, and MCP-1 in the colon of CAC-bearing mice. These cytokines were shown to be involved in the accumulation PMN- and M-MDSCs (17, 34–36). We also detected elevated levels of IL10, an anti-inflammatory cytokine with immunosuppressive effects. We observed that MDSCs accumulate in both periphery (blood, spleen, and BM) and site of inflammation (colon), which led to an immunosuppressive status in CAC-bearing mice. *Ex vivo* coculture experiments showed that MDSCs were able to downregulate TCR CD247 expression and inhibit T-cell proliferation, thus impairing T-cell function.

In addition, we showed that CAC mice exhibit an altered gut microbiota composition when compared with control mice. The CAC mice are characterized by a lower bacterial diversity and elevated levels of Bacteroides, Turicibacter, and Sutterella as compared with control mice. Consistent with our results, previous studies linked elevated levels of Bacteroides and Turicibacter to intestinal inflammation and CAC development (37–39), and several members of the Bacteroides genus were shown to regulate the immune response in a DSS-induced colitis mouse model (40). Elevated levels of Sutterella, which is an IgA degrading genus, have been associated with the pathogenesis of ulcerative colitis and suggested as a biomarker for CAC development (41, 42).

To elucidate the role of the altered microbiota in CAC development and MDSCs function, we employed two approaches to manipulate microbiota composition. In the first approach, GF mice were transplanted with control or CAC bacteria prior to CAC induction. Interestingly, although both groups, the CAC-FMT and control-FMT, were subjected to CAC induction using the same regiment and developed a similar inflammatory response and terminal microbiota composition, the CAC-induced CAC-FMT mice exhibited a significantly higher tumor burden compared with the CAC-induced control-FMT mice. This outcome was accompanied by elevated levels of MDSCs in CAC-FMT CAC-bearing mice, implying a pivotal role of the microbiota in both MDSC accumulation and function. Our findings indicate that the microbiota originating from CAC mice plays a role in the predisposition and promotion of tumor development when intestinal inflammation develops. Moreover, a more in-depth analysis of the correlation between the initial microbiota composition and tumor load revealed that the microbiota not only plays a role in the predisposition to tumor development but can also serve as a predictor of the tumor burden. However, the exact role of all genera involved in predicting the tumor load remains incompletely understood and warrants further investigation. In our second approach, we treated CAC mice with ABX to deplete the microbiota. We demonstrate that ABX treatment of CAC mice at the advanced stage of the disease, which is featured by MDSC accumulation, altered microbiota composition and developing tumors, yielded therapeutic benefits including: a significant reduction in the burden of invading bacteria in the intestine, amelioration of the gut and peripheral inflammation, mitigation of intestinal tissue damage, lowered MDSC levels, and diminished MDSC suppressive functions. Ultimately, this led to a substantial reduction in tumor burden.

Collectively, the results from these two complementary approaches led us to postulate the existence of an intimate cross-talk between MDSCs and bacteria that perpetuates the suppressive activity of MDSCs, creating an inflammatory environment conducive to tumor growth. Indeed, immunofluorescent and FISH analyses unveiled that bacteria invading the colon were situated in close proximity to the recruited MDSCs. Mechanistically, bacteria originating from CAC mice enhanced MDSC suppressive features through various pathways: (i) Increased levels of NO and ROS secretion, (ii) Upregulated activity of arginase, (iii) Elevated expression of the inhibitory checkpoint molecule PD-L1, and (iv) Heightened secretion of inflammatory cytokines. Interestingly, following exposure to the bacteria, M-MDSCs secreted high levels of TNFα, IL6, MCP-1, and IL10, all of which were also elevated in punch biopsies of the colons of CAC mice. While IL10 is generally considered to be an anti-inflammatory cytokine that suppresses inflammation, it has been demonstrated to play a promoting role in tumor development (43). This indicates the dual role of IL10 in CAC: on the one hand, it acts to suppress colon inflammation, but on the other hand, it contributes to tumor growth. It has been documented that MDSCs are a major source of IL10 in the inflamed colon, mediating epithelial transformation (44). Thus, it is plausible that the heightened levels of IL10 detected in CAC mice originate from the recruited MDSCs following their interaction with the bacteria, consequently shifting the function of IL10 an inflammation-resolving cytokine to a tumor-promoting one. In addition, our findings revealed that M-MDSCs became more suppressive toward impairing T-cell function following interaction with bacteria. We further identified that CAC-derived bacteria altered the differentiation pathway of MDSC. On one hand, the bacteria arrested M-MDSCs in their immature and immunosuppressive state, inhibiting their terminal differentiation to mature myeloid cells mediated by GMCSF and MCSF. On the other hand, the bacteria skewed MDSCs toward an M2-like phenotype. M2 macrophages are considered protumorigenic as they support angiogenesis, tissue remodeling, and suppress antitumor immune responses (45). Supporting our results, studies have shown that bacterial and fungal components such as lipopolysaccharide, flagellin, zymosan, and secreted extracellular membrane vesicles from commensal bacteria induce expansion, activation, and differentiation of MDSCs (46–50).

We also report that colorectal tumors of CAC mice are colonized with bacteria belonging to the Turicibacter, Sutterella, Bacteroides genera, correlating with those dominating the fecal samples. However, further research is needed to unravel the role of specific bacteria that colonize colorectal tumors and the signaling pathways activated. This will provide a better understanding of the cellular and molecular mechanisms by which extratumoral and intratumoral bacteria induce immunosuppression in the tumor microenvironment.

In conclusion, our findings offer an in-depth understanding of the immunosuppressive environment induced by the cross-talk between the intestinal microbiota and MDSCs during colitis, underscoring its contribution to colon cancer development. The results presented herein suggest that manipulating the gut microbiota to disrupt the bacteria–MDSC interaction, could serve as a modality to reduce intestinal inflammation and the persistent activation of MDSCs. This approach is expected to alleviate immunosuppression, a major obstacle in various anticancer responses and treatments, potentially leading to a rejuvenated immune environment that enhances the success rates of immune- and chemo-based therapies. A deeper understanding of the tumor microbiota's composition and its role in the tumor microenvironment and immunosuppression may pave the way for the development of more specific and successful combinatorial treatments.

## Supplementary Material

Figure S1 Supplementary Figure S1 shows established tumors and colonic inflammation in CAC mice as compared to control mice.

Figure S2 Supplementary Figure S2 shows a pronounced inflammation in CAC vs. control mice as indicated by elevated cytokine levels in colon punch biopsies and the recruitment of MDSCs in colonic tissues.

Figure S3 Supplementary Figure S3 shows the differences between the MDSC sub-populations in their suppressive activity/

Figure S4 Supplementary Figure S4 shows the microbiota composition in the pools from CAC and control mice used for FMT and the changes in relative abundance of unique bacteria following disease progression.

Figure S5 Supplementary Figure S5 shows the beneficial ABX treatment effects on CAC mice with established tumors and colonic inflammation as indicated by lessening colonic inflammation and leading to tumor regression as compared to non-ABX treated CAC mice.

Figure S6 Supplementary Figure S6 shows the positive effects of ABX treatment on dysbiosis in CAC mice, leading to reduced levels of bacteria associated with CAC. This is accompanied by a significant reduction in colonic invading bacteria compared to non-ABX treated CAC mice.

Figure S7 Supplementary Figure S7 shows that some cytokine levels in colon punch biopsies, which are elevated in CAC vs. control mice are unaffected by ABX treatment.

Figure S8 Supplementary Figure S8 shows that fecal dysbiotic bacteria from CAC mice had a more pronounced impact on the morphology of PMN-MDSCs compared to M-MDSCs.
