# Supplementary material for: The Cross-talk Between Intestinal Microbiota and MDSCs Fuels Colitis-associated Cancer Development
Source: Cancer Res Commun. 2024 Apr 15;4(4):1063–81. doi: 10.1158/2767-9764.CRC-23-0421 (PMC11017962; doi:10.1158/2767-9764.CRC-23-0421)
Supplement: Figure S1 — Supplementary Figure S1 shows established tumors and colonic inflammation in CAC mice as compared to control mice. [file crc-23-0421-s01.pptx]

## Slide 1
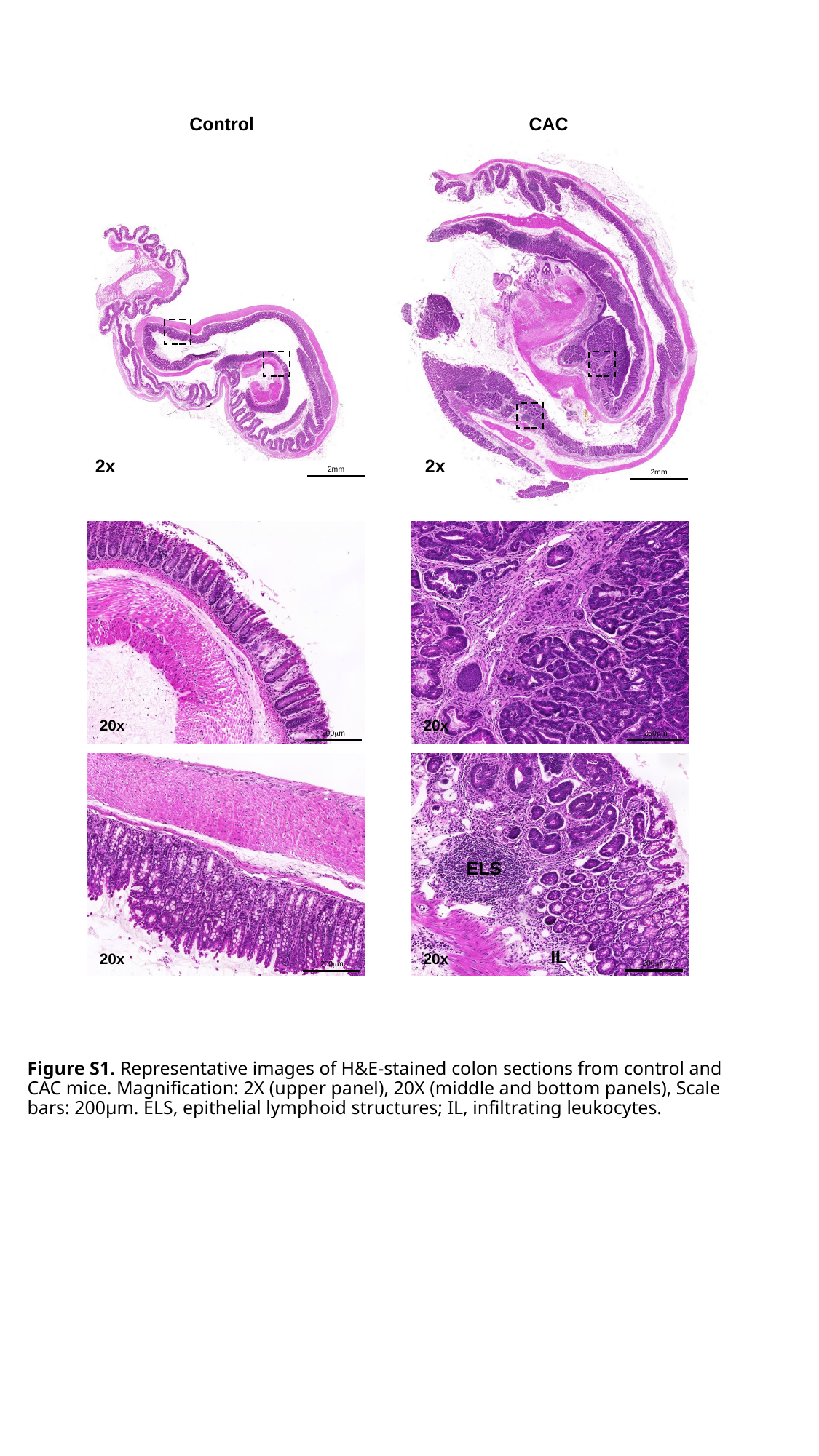

Control
CAC
2x
2x
2mm
2mm
20x
20x
200mm
200mm
ELS
IL
20x
20x
200mm
200mm
Figure S1. Representative images of H&E-stained colon sections from control and CAC mice. Magnification: 2X (upper panel), 20X (middle and bottom panels), Scale bars: 200µm. ELS, epithelial lymphoid structures; IL, infiltrating leukocytes.
