# Supplementary material for: The Cross-talk Between Intestinal Microbiota and MDSCs Fuels Colitis-associated Cancer Development
Source: Cancer Res Commun. 2024 Apr 15;4(4):1063–81. doi: 10.1158/2767-9764.CRC-23-0421 (PMC11017962; doi:10.1158/2767-9764.CRC-23-0421)
Supplement: Figure S2 — Supplementary Figure S2 shows a pronounced inflammation in CAC vs. control mice as indicated by elevated cytokine levels in colon punch biopsies and the recruitment of MDSCs in colonic tissues. [file crc-23-0421-s02.pptx]

## Slide 1
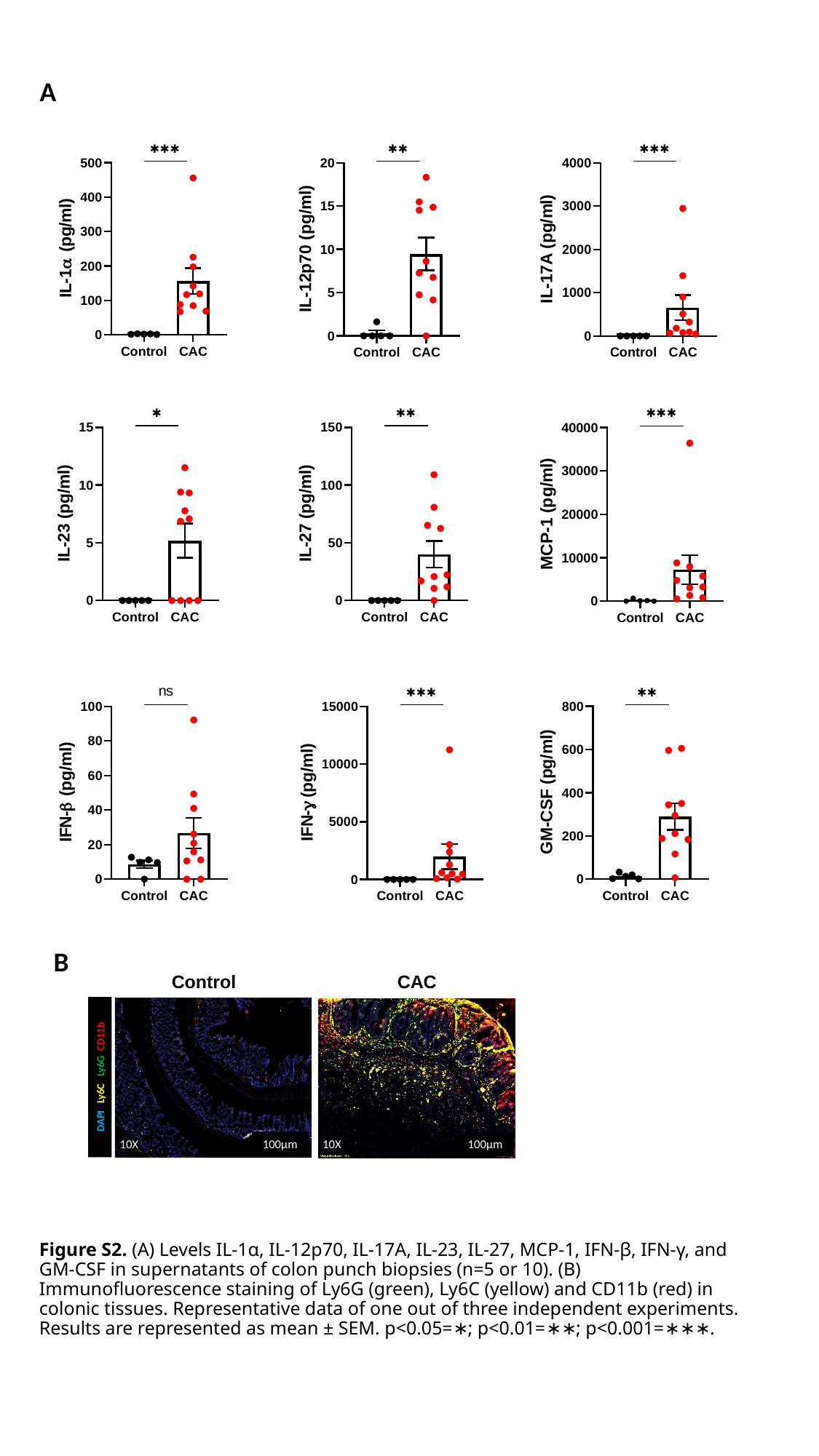

A
b
Control
CAC
DAPI Ly6C Ly6G CD11b
10X
100µm
10X
100µm
10X
Figure S2. (A) Levels IL-1α, IL-12p70, IL-17A, IL-23, IL-27, MCP-1, IFN-β, IFN-γ, and GM-CSF in supernatants of colon punch biopsies (n=5 or 10). (B) Immunofluorescence staining of Ly6G (green), Ly6C (yellow) and CD11b (red) in colonic tissues. Representative data of one out of three independent experiments. Results are represented as mean ± SEM. p<0.05=∗; p<0.01=∗∗; p<0.001=∗∗∗.
