# Supplementary material for: The Cross-talk Between Intestinal Microbiota and MDSCs Fuels Colitis-associated Cancer Development
Source: Cancer Res Commun. 2024 Apr 15;4(4):1063–81. doi: 10.1158/2767-9764.CRC-23-0421 (PMC11017962; doi:10.1158/2767-9764.CRC-23-0421)
Supplement: Figure S3 — Supplementary Figure S3 shows the differences between the MDSC sub-populations in their suppressive activity/ [file crc-23-0421-s03.pptx]

## Slide 1
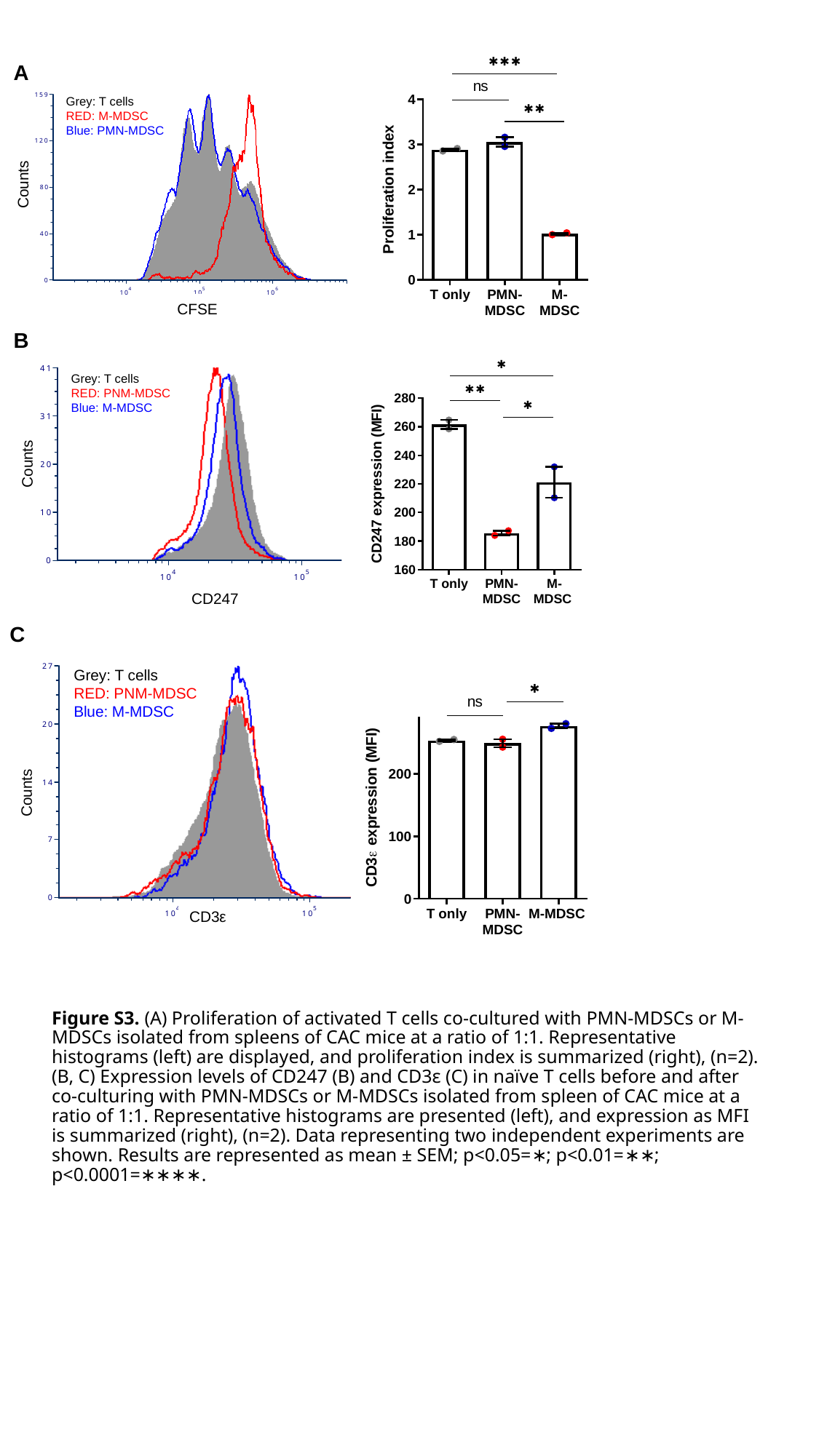

A
Grey: T cells
RED: M-MDSC
Blue: PMN-MDSC
Counts
CFSE
B
Grey: T cells
RED: PNM-MDSC
Blue: M-MDSC
CD247
Counts
C
CD3ε
Grey: T cells
RED: PNM-MDSC
Blue: M-MDSC
Counts
Figure S3. (A) Proliferation of activated T cells co-cultured with PMN-MDSCs or M-MDSCs isolated from spleens of CAC mice at a ratio of 1:1. Representative histograms (left) are displayed, and proliferation index is summarized (right), (n=2). (B, C) Expression levels of CD247 (B) and CD3ε (C) in naïve T cells before and after co-culturing with PMN-MDSCs or M-MDSCs isolated from spleen of CAC mice at a ratio of 1:1. Representative histograms are presented (left), and expression as MFI is summarized (right), (n=2). Data representing two independent experiments are shown. Results are represented as mean ± SEM; p<0.05=∗; p<0.01=∗∗; p<0.0001=∗∗∗∗.
