# Supplementary material for: The Cross-talk Between Intestinal Microbiota and MDSCs Fuels Colitis-associated Cancer Development
Source: Cancer Res Commun. 2024 Apr 15;4(4):1063–81. doi: 10.1158/2767-9764.CRC-23-0421 (PMC11017962; doi:10.1158/2767-9764.CRC-23-0421)
Supplement: Figure S4 — Supplementary Figure S4 shows the microbiota composition in the pools from CAC and control mice used for FMT and the changes in relative abundance of unique bacteria following disease progression. [file crc-23-0421-s04.pptx]

## Slide 1
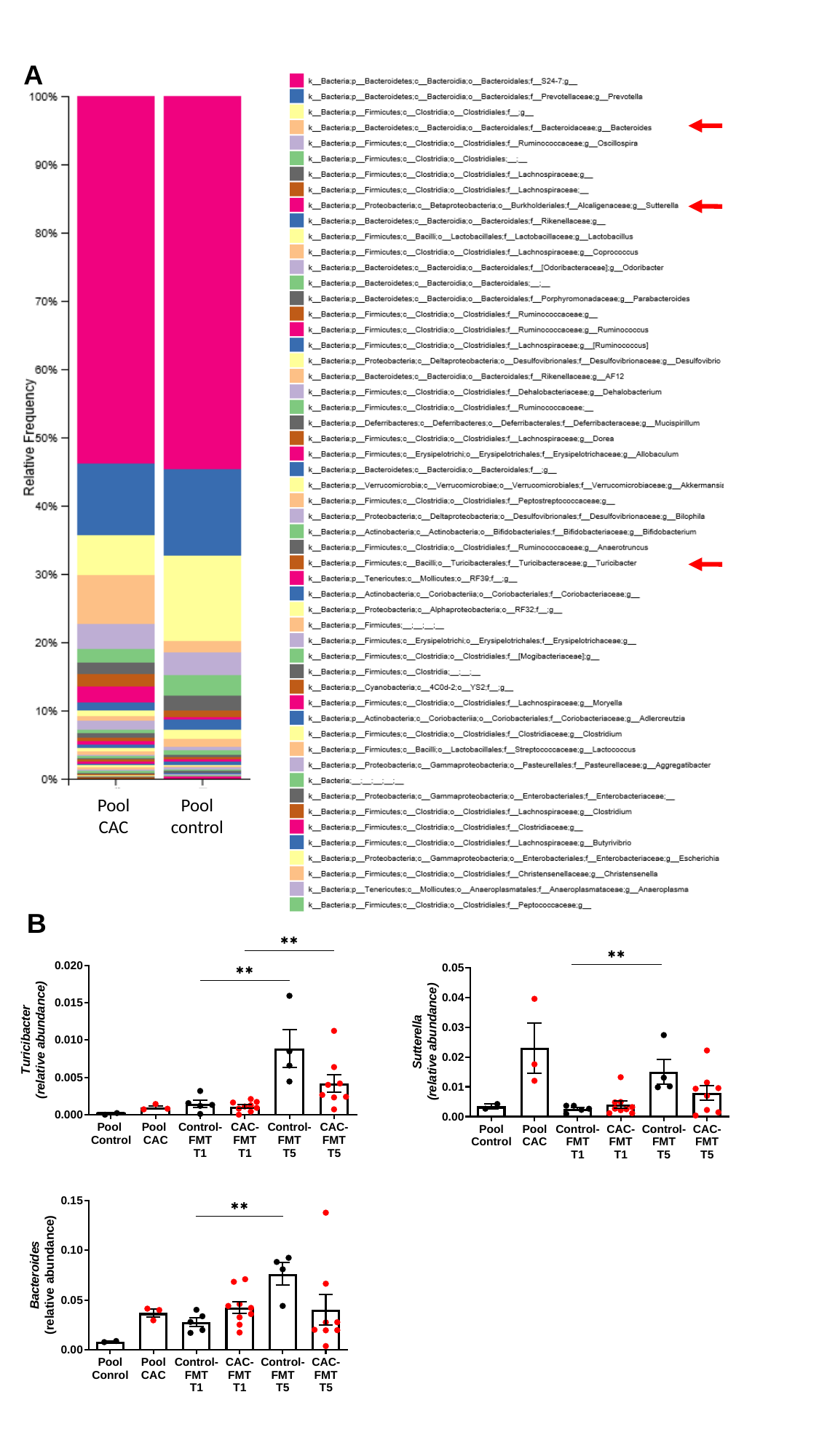

A
Pool
CAC
Pool
control
B

## Slide 2
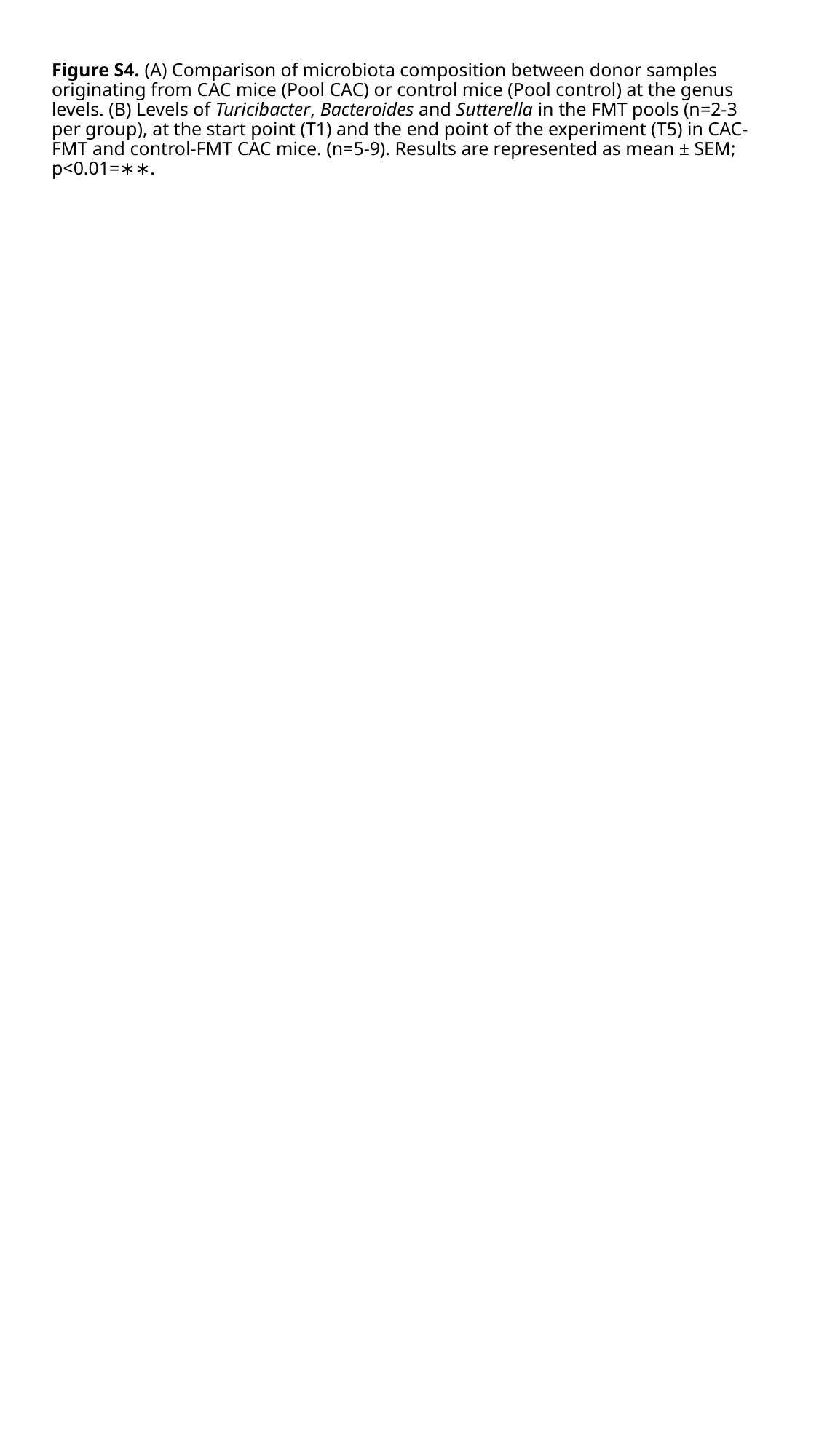

Figure S4. (A) Comparison of microbiota composition between donor samples originating from CAC mice (Pool CAC) or control mice (Pool control) at the genus levels. (B) Levels of Turicibacter, Bacteroides and Sutterella in the FMT pools (n=2-3 per group), at the start point (T1) and the end point of the experiment (T5) in CAC-FMT and control-FMT CAC mice. (n=5-9). Results are represented as mean ± SEM; p<0.01=∗∗.
