# Supplementary material for: The Cross-talk Between Intestinal Microbiota and MDSCs Fuels Colitis-associated Cancer Development
Source: Cancer Res Commun. 2024 Apr 15;4(4):1063–81. doi: 10.1158/2767-9764.CRC-23-0421 (PMC11017962; doi:10.1158/2767-9764.CRC-23-0421)
Supplement: Figure S5 — Supplementary Figure S5 shows the beneficial ABX treatment effects on CAC mice with established tumors and colonic inflammation as indicated by lessening colonic inflammation and leading to tumor regression as compared to non-ABX treated CAC mice. [file crc-23-0421-s05.pptx]

## Slide 1
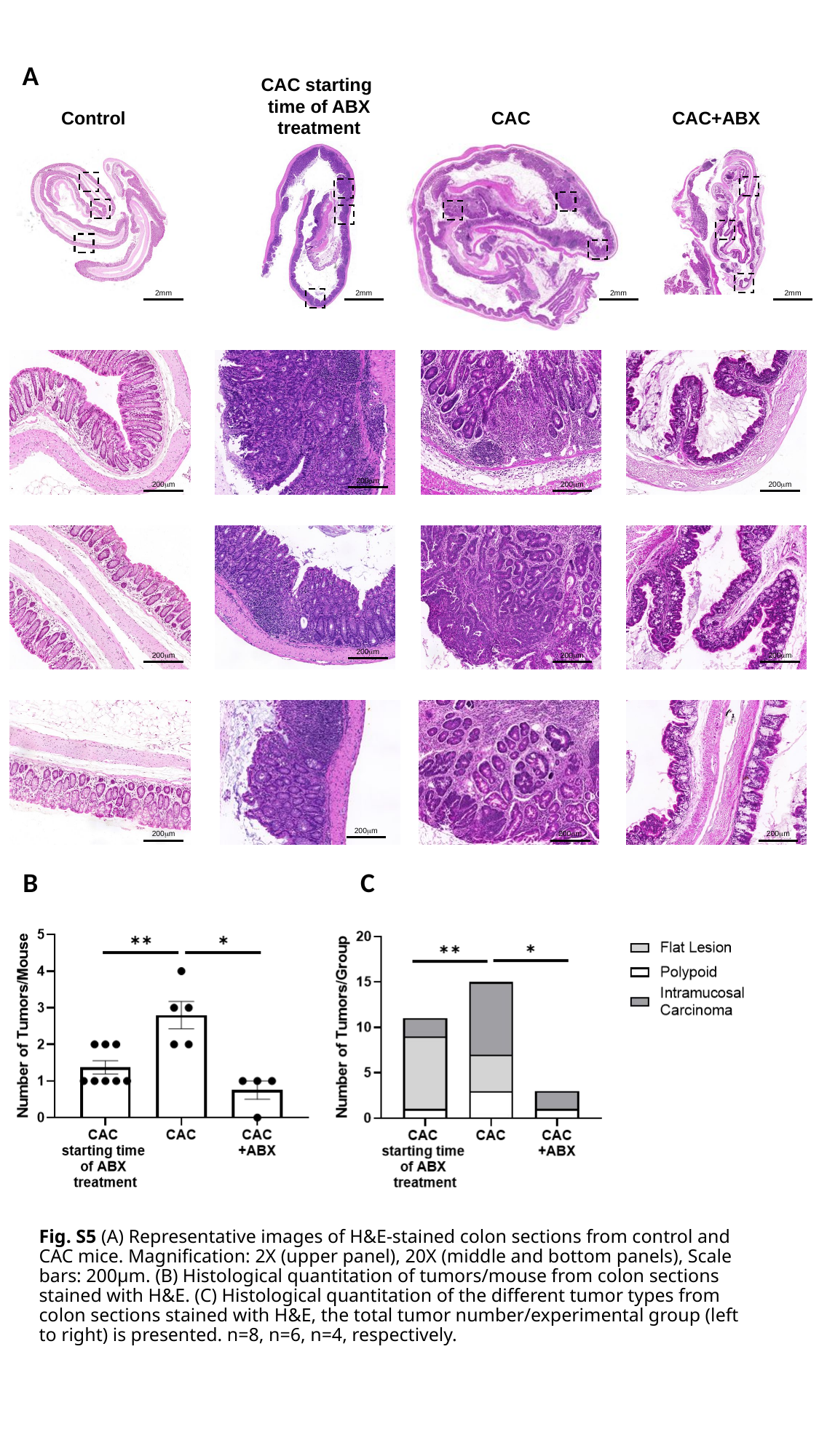

A
CAC starting time of ABX
treatment
Control
CAC
CAC+ABX
2mm
2mm
2mm
2mm
200mm
200mm
200mm
200mm
200mm
200mm
200mm
200mm
200mm
200mm
200mm
200mm
B
C
Fig. S5 (A) Representative images of H&E-stained colon sections from control and CAC mice. Magnification: 2X (upper panel), 20X (middle and bottom panels), Scale bars: 200µm. (B) Histological quantitation of tumors/mouse from colon sections stained with H&E. (C) Histological quantitation of the different tumor types from colon sections stained with H&E, the total tumor number/experimental group (left to right) is presented. n=8, n=6, n=4, respectively.
