# Supplementary material for: The Cross-talk Between Intestinal Microbiota and MDSCs Fuels Colitis-associated Cancer Development
Source: Cancer Res Commun. 2024 Apr 15;4(4):1063–81. doi: 10.1158/2767-9764.CRC-23-0421 (PMC11017962; doi:10.1158/2767-9764.CRC-23-0421)
Supplement: Figure S6 — Supplementary Figure S6 shows the positive effects of ABX treatment on dysbiosis in CAC mice, leading to reduced levels of bacteria associated with CAC. This is accompanied by a significant reduction in colonic invading bacteria compared to non-ABX treated CAC mice. [file crc-23-0421-s06.pptx]

## Slide 1
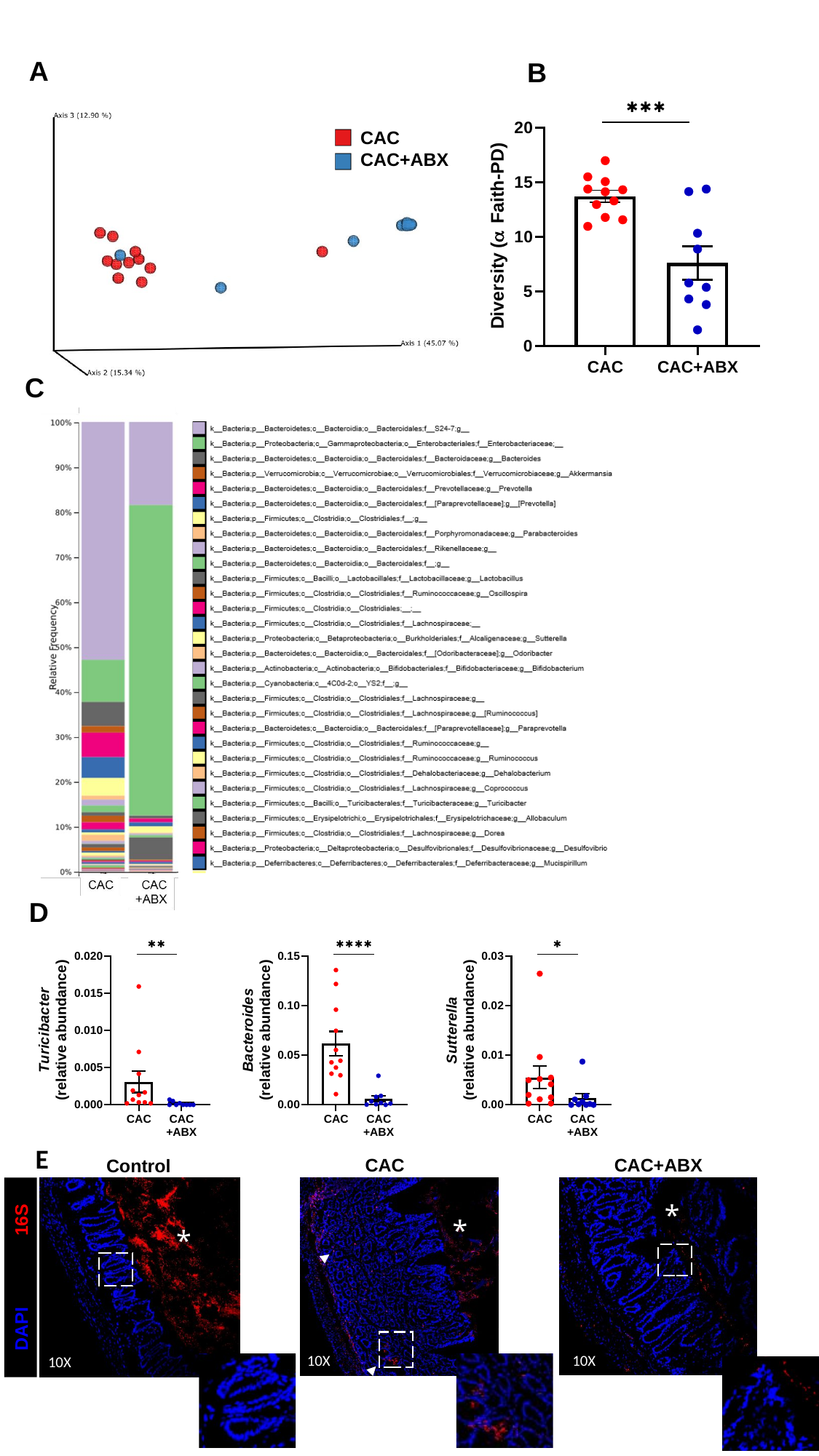

A
B
CAC
CAC+ABX
C
D
E
CAC+ABX
CAC
Control
*
*
*
*
DAPI 16S
10X
10X
10X
10X

## Slide 2
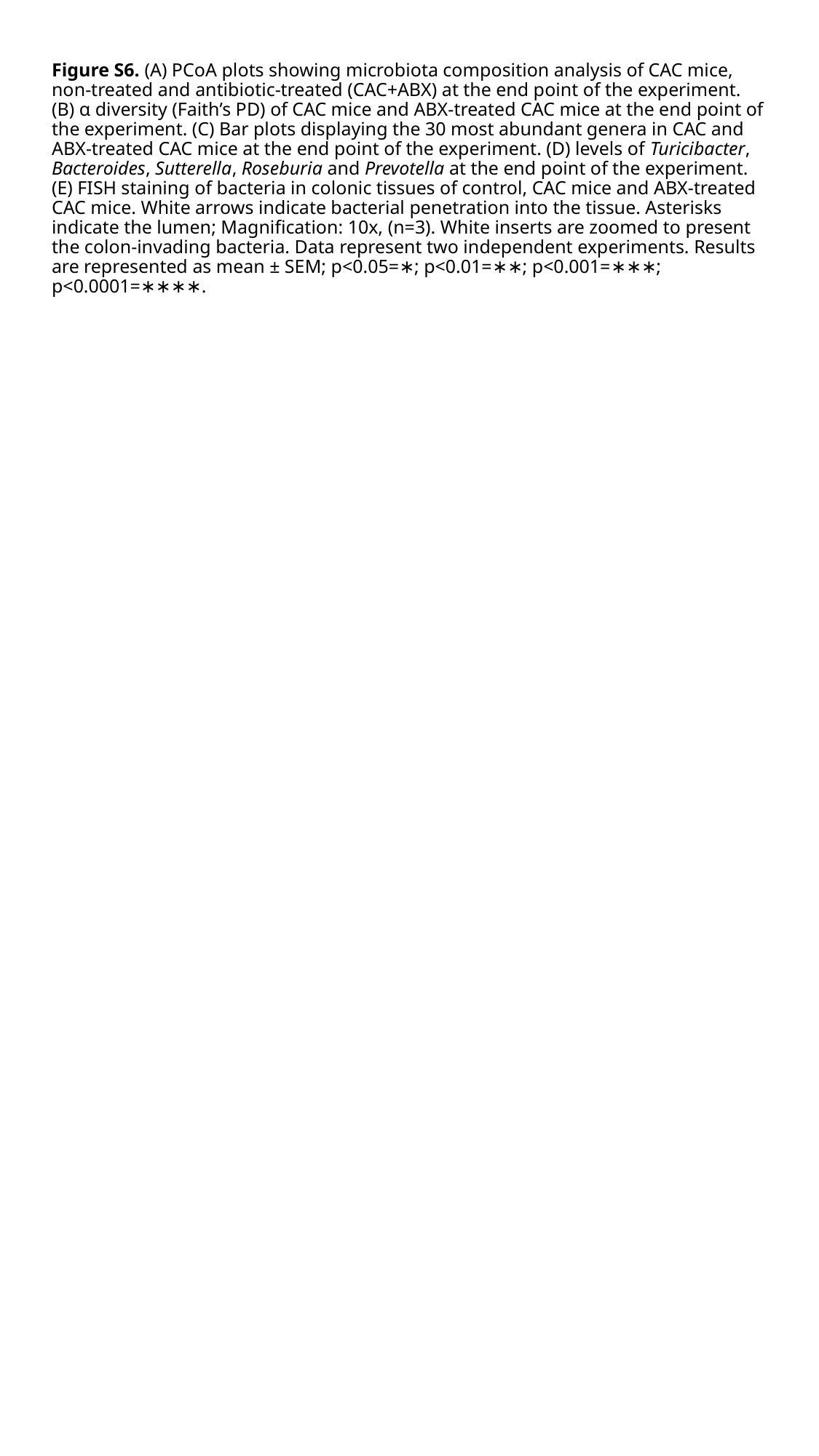

Figure S6. (A) PCoA plots showing microbiota composition analysis of CAC mice, non-treated and antibiotic-treated (CAC+ABX) at the end point of the experiment. (B) α diversity (Faith’s PD) of CAC mice and ABX-treated CAC mice at the end point of the experiment. (C) Bar plots displaying the 30 most abundant genera in CAC and ABX-treated CAC mice at the end point of the experiment. (D) levels of Turicibacter, Bacteroides, Sutterella, Roseburia and Prevotella at the end point of the experiment. (E) FISH staining of bacteria in colonic tissues of control, CAC mice and ABX-treated CAC mice. White arrows indicate bacterial penetration into the tissue. Asterisks indicate the lumen; Magnification: 10x, (n=3). White inserts are zoomed to present the colon-invading bacteria. Data represent two independent experiments. Results are represented as mean ± SEM; p<0.05=∗; p<0.01=∗∗; p<0.001=∗∗∗; p<0.0001=∗∗∗∗.
