# Supplementary material for: The Cross-talk Between Intestinal Microbiota and MDSCs Fuels Colitis-associated Cancer Development
Source: Cancer Res Commun. 2024 Apr 15;4(4):1063–81. doi: 10.1158/2767-9764.CRC-23-0421 (PMC11017962; doi:10.1158/2767-9764.CRC-23-0421)
Supplement: Figure S7 — Supplementary Figure S7 shows that some cytokine levels in colon punch biopsies, which are elevated in CAC vs. control mice are unaffected by ABX treatment. [file crc-23-0421-s07.pptx]

## Slide 1
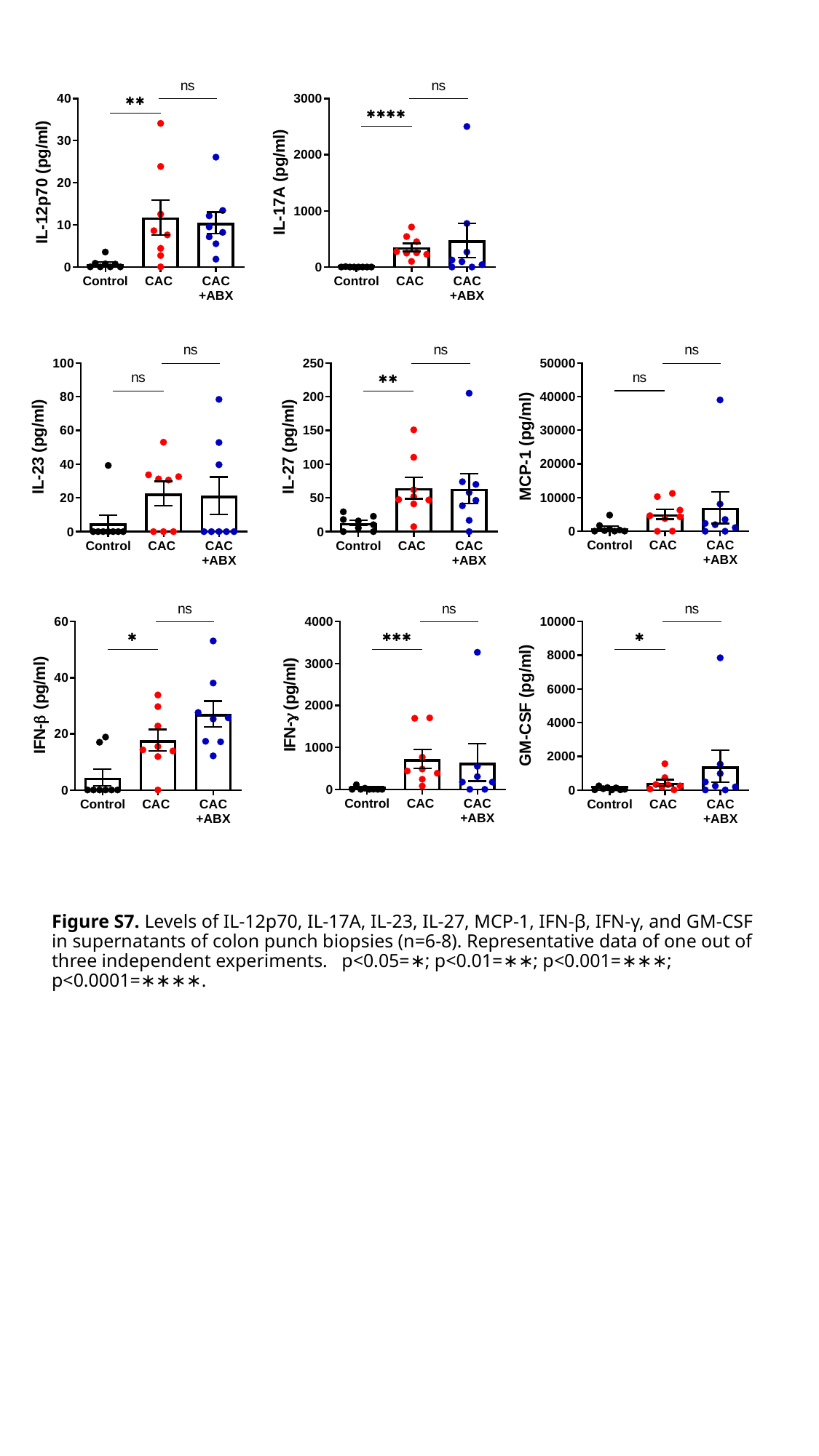

Figure S7. Levels of IL-12p70, IL-17A, IL-23, IL-27, MCP-1, IFN-β, IFN-γ, and GM-CSF in supernatants of colon punch biopsies (n=6-8). Representative data of one out of three independent experiments. p<0.05=∗; p<0.01=∗∗; p<0.001=∗∗∗; p<0.0001=∗∗∗∗.
