# Supplementary material for: The Cross-talk Between Intestinal Microbiota and MDSCs Fuels Colitis-associated Cancer Development
Source: Cancer Res Commun. 2024 Apr 15;4(4):1063–81. doi: 10.1158/2767-9764.CRC-23-0421 (PMC11017962; doi:10.1158/2767-9764.CRC-23-0421)
Supplement: Figure S8 — Supplementary Figure S8 shows that fecal dysbiotic bacteria from CAC mice had a more pronounced impact on the morphology of PMN-MDSCs compared to M-MDSCs. [file crc-23-0421-s08.pptx]

## Slide 1
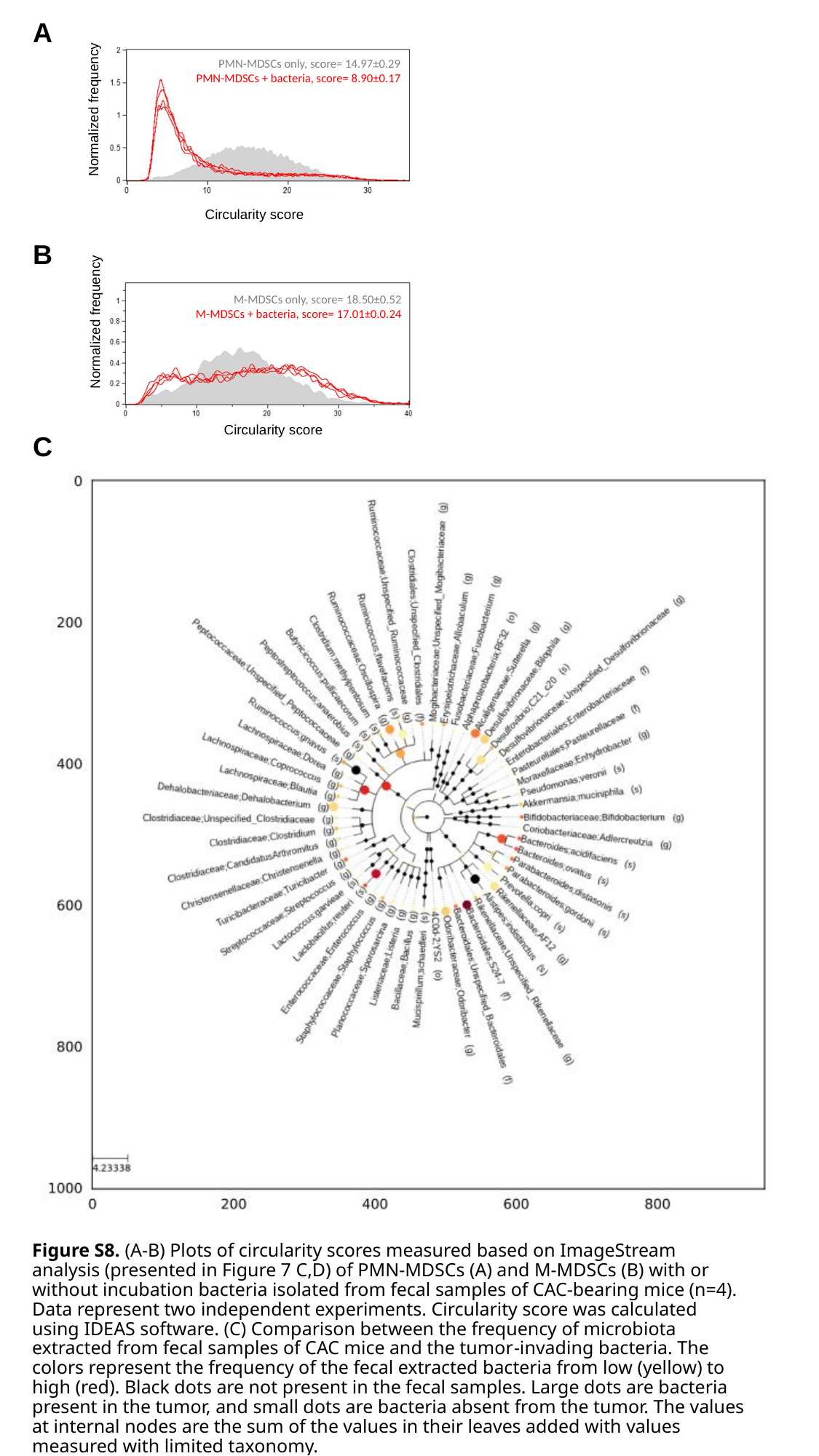

A
PMN-MDSCs only, score= 14.97±0.29
PMN-MDSCs + bacteria, score= 8.90±0.17
Normalized frequency
Circularity score
B
M-MDSCs only, score= 18.50±0.52
M-MDSCs + bacteria, score= 17.01±0.0.24
Normalized frequency
Circularity score
C
Figure S8. (A-B) Plots of circularity scores measured based on ImageStream analysis (presented in Figure 7 C,D) of PMN-MDSCs (A) and M-MDSCs (B) with or without incubation bacteria isolated from fecal samples of CAC-bearing mice (n=4). Data represent two independent experiments. Circularity score was calculated using IDEAS software. (C) Comparison between the frequency of microbiota extracted from fecal samples of CAC mice and the tumor-invading bacteria. The colors represent the frequency of the fecal extracted bacteria from low (yellow) to high (red). Black dots are not present in the fecal samples. Large dots are bacteria present in the tumor, and small dots are bacteria absent from the tumor. The values at internal nodes are the sum of the values in their leaves added with values measured with limited taxonomy.
